# CREB-SEC61G feedback loop sustains enhanced autophagy and boosts proliferation in PDAC

**DOI:** 10.1038/s41419-026-08915-7

**Published:** 2026-05-29

**Authors:** Xiao Wu, Xinyan Wu, Yihang Nan, Jianhui Li, Lei Zhuang, Wengang Shan, Song Ge, Kun Tong, Bin Zhang, Qingwei Song, Xuhao Ni

**Affiliations:** 1 Department of General Surgery, The Affiliated Hospital of Xuzhou Medical University, Xuzhou, China; 2 Department of Pathophysiology, Zhongshan School of Medicine, Sun Yat-sen University, Guangzhou, China; 3 Department of Hepatobiliary Surgery, The Second Affiliated Hospital of Guangzhou Medical University, Guangzhou, China; 4 Department of Anesthesia, The Affiliated Hospital of Xuzhou Medical University, Xuzhou, China; 5 Department of Hepatobiliary Surgery, The First Affiliated Hospital of Anhui Medical University, Hefei, China

**Keywords:** Pancreatic cancer, Autophagy

## Abstract

Pancreatic ductal adenocarcinoma (PDAC) is one of the most lethal malignancies, with rising incidence and mortality rates, in which autophagy plays a pivotal role in promoting tumor survival. Although SEC61G has been reported to be associated with poor outcomes in patients with PDAC, its underlying mechanism remains largely unclear. In the present study, we found that SEC61G was overexpressed in PDAC and correlated with an unfavorable patient prognosis. Further experiments demonstrated the pro-proliferation efficacy of SEC61G in PDAC progression both in vitro and in vivo. Mechanistically, SEC61G enhanced cellular autophagy in PDAC in a Ca^2+^ leakage-dependent manner, which could be reversed by thapsigargin treatment. Interestingly, we unveiled that the elevated intracellular Ca²⁺ concentration mediated by SEC61G could activate CREB, phosphorylated CREB bound to the SEC61G promoter and consequently enhanced SEC61G expression. This positive feedback loop may account for the persistent activation of autophagy in PDAC. Our findings highlight the potential of SEC61G as a prognostic biomarker and a therapeutic target candidate for combating PDAC.

## Introduction

Pancreatic ductal adenocarcinoma (PDAC) is one of the most lethal malignancies with an alarming rise in both incidence and mortality rates, ranking it among the leading causes of cancer-related deaths worldwide [1, 2]. Notably, only about 20% of patients are eligible for surgical resection at the time of diagnosis, primarily due to the asymptomatic nature of PDAC during its early stages and the lack of reliable early detection biomarkers [3, 4]. The hallmark feature of PDAC is its remarkable ability to survive and proliferate within the nutrient-deprived and hypoxic tumor microenvironment. This resilience largely depends on tumor nutrient-scavenging mechanisms such as macropinocytosis [5] and autophagy [6]. Therefore, therapeutic strategies aimed at “starving” tumor cells—for instance, by inhibiting autophagy—may represent a promising approach to combat PDAC in the future.

Autophagy plays an essential role in maintaining cellular homeostasis and integrity by degrading and recycling intracellular components, including damaged proteins, organelles and cellular debris [7, 8]. In cancer, autophagy functions as a double-edged sword, with either tumor-suppressive or tumor-promoting effects, depending on tumor type, stage, and molecular context [9]. In PDAC, autophagy facilitates tumor adaptation to harsh environmental conditions such as nutrient deprivation, hypoxia, and immune surveillance [7]. Elevated autophagic activity has been consistently observed in PDAC cell lines and primary tumors [10]. However, the mechanisms underlying the sustained activation of autophagy in PDAC remain poorly understood.

The SEC61 translocon complex is composed of three highly conserved subunits—SEC61A, SEC61B, and SEC61G—and plays an indispensable role in protein folding, modification and translocation, as well as in the activation of the unfolded protein response [11]. Additionally, the SEC61 complex has been shown to serve as a channel to enable calcium (Ca^2+^) leakage from endoplasmic reticulum (ER) into cytosol [12]. Recent studies have demonstrated that dysregulation of SEC61G is associated with tumor progression and poor prognosis in several malignancies, including brain [13], lung [14], breast [15], liver [16] and colon cancers [17].

In the present study, we found that SEC61G was highly expressed in PDAC tissues and predicted poor survival outcomes in PDAC patients. Moreover, SEC61G promoted tumor cell proliferation both in vitro and in vivo. Mechanistically, SEC61G enhanced autophagy in PDAC cells. Further investigation unveiled that the CREB-SEC61G positive feedback loop sustained Ca^2+^ leakage into cytosol and thereby maintained persistent autophagic activity in PDAC, which could be blocked by depleting intracellular Ca²⁺ storage.

## Materials and methods

### Patients

70 PDAC patients, who were diagnosed by histology and underwent pancreatectomy at the Affiliated Hospital of Xuzhou Medical University (Xuzhou, China), were involved in this study, which was approved by the Ethical Committee of the Affiliated Hospital of Xuzhou Medical University. Informed consent was obtained from all patients. The clinicopathological characteristics of those patients are shown in the supplementary tables. The sample proteins were harvested using the minute^TM^ total protein extraction Kit (Invent, SD-001) as previously described, and subsequently subjected to a western blot assay. As well, total RNA was extracted with TRIzol (Thermo) and then subject to RT-qPCR to determine the expression of indicated genes with specific primers. Paraffin-embedded specimens from these 70 patients were used for subsequent HE and IHC assays.

### Animal experiments

As previous described [18], NCG mice (aged 4–6 weeks) were purchased from the GemPharmatech Co., Ltd (Nanjing, China) and maintained under specific pathogen-free conditions. Animal protocols were approved by the Animal Care and Use Committee of Sun Yat-Sen University. Mice were injected subcutaneously with 2×10^6^ Patu-8988 T cells. Five biological replicates were used for each treatment group. The tumors were measured every 5 days. The formula for tumor volume calculation was 0.5×length×width×height. Tumor-bearing mice were sacrificed on the 30th day, and the tumors were harvested for further study. To verify that thapsigargin could rescue the pro-tumor effect of SEC61G overexpression in vivo, thapsigargin (MCE, HY-13433) was purchased and dissolved in dimethyl sulfoxide (DMSO) and intraperitoneally injected into mice at 0.25 mg/kg twice a week when tumors were palpable, around 5 days after implantation.

### Bioinformatic analysis and online platforms

**Fig. 1:**
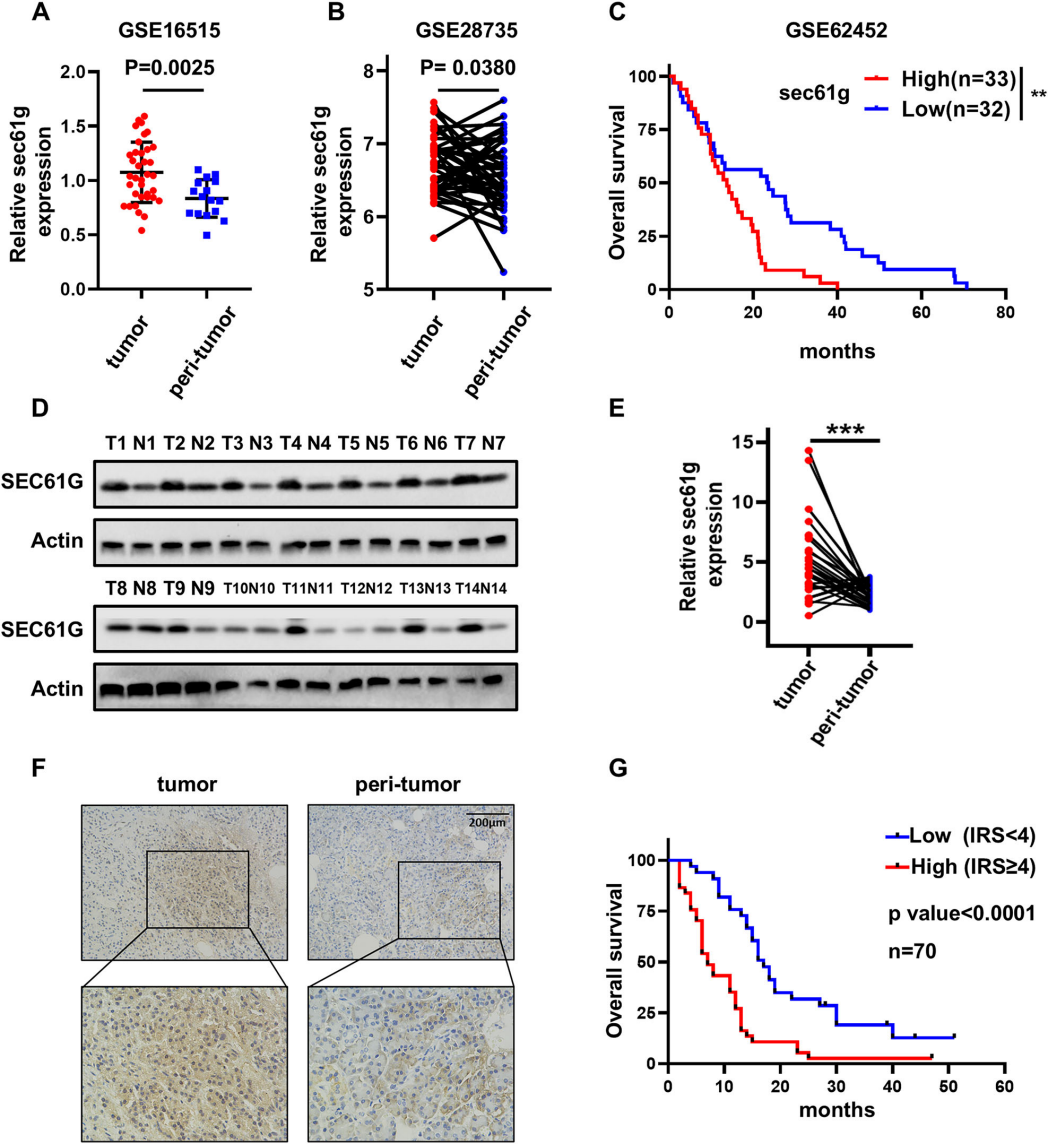
SEC61G is upregulated in PDAC tissues and correlates with an unfavorable prognosis. **A, B** Analysis of sec61g expression in online databases (GSE16515, unpaired data. n (tumor) =36, n (peri-tumor) =16; GSE28735, a paired data. *n* = 45.), t test; (**C**). Overall survival based on sec61g expression in GSE62452 database. Kaplan–Meier test, **p < 0.01. **D** Expression of SEC61G was determined by western blot in fourteen paired primary pancreatic cancer tissues (T) and the matched adjacent nontumor tissues (N). **E** Relative expression of sec61g of PDAC and paired peri-tumor issues was determined by RT-qPCR from 29 patients with PDAC, paired T test, ***p < 0.001. **F** Representative IHC image of SEC61G in PDAC and paired peri-tumor tissues using PDAC tissue microarray (TMA), Scale bar shown is 200 μm. (**G**). Overall survival based on SEC61G expression in TAM. Kaplan–Meier test.

Three gene expression profiles (GSE16515, GSE28735, GSE62452) were downloaded from the Gene Expression Omnibus (GEO) database, the expression of sec61a, sec61b.sec61g and lc3b in tumors as well as patient survival was extracted and used to 1) compare the expression of indicated gene between tumor and peri-tumor issue, like Figs. 1A, B and S1A, B; 2) compare the survival between patients with different expression of indicated gene, like Figs. 1C and S1C; 3) analyze the correlation between sec61g and lc3b at the mRNA level (Fig. S4C). On the other hand, the online JASPAR database (https://jaspar.elixir.no/) was utilized to predict the potential binding points of CREB within the promoter of sec61g.

### RNA sequencing analysis

An RNA-seq assay between Patu-8988t with reduced SEC61G or not was performed. As previously described [19], the gene count data were normalized using the relative log expression method, which accounts for RNA-seq library size differences. A gene was defined as DEGs if the Benjamini–Hochberg adjusted *p*-value was less than 0.05 and fold changes were greater than or equal to 2. A gene ontology (GO) functional enrichment analysis was conducted using the R packages GOSeq (version 1.10.0) and topGO (version 2.10.0).14 The DEGs were significantly enriched (in GO terms) when their Bonferroni-corrected *p*-values were less than or equal to 0.05. The gene set enrichment analysis (GSEA) presented in this study was performed using Java GSEA.15 The alignment background was referred to as the Molecular Signatures Database v7.5. Related RNA-Seq data have been deposited in the SRA database.

### Autophagic flux analysis

PDAC cells were infected with mCherry-GFP-LC3 (biotime, China), according to the manufacturer’s instructions. Images were captured with fluorescence microscopy (Carl Zeiss). Green or red fluorescence was used to monitor the progression of autophagic flux with different expression of SEC61G.

### Transmission electron microscopy

Cells with distinct SEC61G expression were collected and fixed with 2% glutaraldehyde overnight. After twice PBS washing, samples were postfixed with OsO4 (1%) for 2 h at RT. A series of graded ethanol and acetone was used to dehydrate samples, which were then placed in acetone/Epon 812 overnight. After 48 h of embedding at 60 °C, 60–80 nm sections were prepared and then subjected to uranyl acetate (15 min) and then Reynold’s lead citrate (15 min) staining. Pictures were taken with TEM (HITACHI, HT7700, Japan).

### Chromatin immunoprecipitation

Patu-8988 T cells were cross-linked with formaldehyde (1%, v/v) for 10 min at RT, which was terminated with glycine (0.125 M). After lysing on ice, Chromatin DNA was subject to sonication to obtain nucleic acid fragments with a length of ~500 bp, which were incubated with anti-CREB antibody. CREB-binding fragments were enriched with protein G-agarose (Santa Cruz). RT-qPCR with precipitated DNA was carried out to determine the putative binding sites of CREB in the promoter of SEC61G.

### Dual luciferase reporter assay

As previously described [20], the indicated promoter of sec61g (Fig. 6F) without mutation or not was constructed and inserted into pGL3-basic to form the reporter plasmids. HEK 293t cells were co-transfected with individual reporter plasmid and control pRL Renilla vector for 48 h. The bioluminescence intensity of Renilla and luciferase was detected using the Dual-Luciferase Reporter Assay System (Promega, #E1910).

### Statistical analysis

As previously described [21], values were presented as the mean ± standard deviation (SD) and statistical analysis was performed using SPSS software 24.0 (USA) or GraphPad Prism 8.0 (USA). Statistical differences between two groups were determined using an unpaired, two-tailed Student’s test. Chi-Square test was used for testing the differences between categorical variables. The survival curves were obtained by the Kaplan–Meier method and compared using the log-rank test. Linear regression analysis was used to determine the correlation between sec61g and lc3b expression levels in human specimens. In general, *P* value < 0.05 were considered significant and are indicated as follows: *P < 0.05, **P < 0.01, ***P < 0.001, ****P < 0.0001, ns: no significance.

## Results

### SEC61G is overexpressed in PDAC and correlates with an unfavorable prognosis

Although the essential role of the SEC61 complex in tumor biology has been demonstrated in several studies [12, 13], its specific function and mechanism in PDAC remain to be fully understood.

The SEC61 complex consists of three core subunits, SEC61A, SEC61B and SEC61G, the expression of which was determined using two Gene Expression Omnibus datasets (GEO16515, unpaired data; GEO28735, paired data). As shown in Fig. S1A and B, sec61a was comparable between tumor and normal pancreatic tissue, whereas sec61b expression was lower in tumors in both datasets. On the other hand, sec61g expression was significantly elevated in PDAC tissues in both datasets (Fig.1A, B). Furthermore, analysis of the GSE62452 dataset revealed that sec61b expression showed no significant association with patient survival (Fig. S2C), while patients with higher sec61g expression showed poorer overall survival (Fig. 1C), despite comparable sec61g expression across different TNM stages (Fig. S1D).

To validate these findings, PDAC tumors and paired para-tumor tissues were collected for western blotting, RT-qPCR and immunohistochemical (IHC) analyses. Consistently, SEC61G was highly expressed in PDAC at both the mRNA (Fig. 1E) and protein levels (Figs. 1D, F and S1E). Importantly, patients with higher SEC61G expression, determined by IHC, exhibited worse overall survival than those with lower expression (Fig. 1G). Both univariate and multivariate analyses confirmed that high SEC61G expression was independently associated with poor prognosis in PDAC patients, implying that SEC61G might serve as a potential indicator for predicting prognosis in patients suffering from PDAC (supplementary Tables). Collectively, these results indicate that SEC61G is upregulated in PDAC and predicts unfavorable survival outcomes.

### SEC61G promotes cellular proliferation and migration in vitro

**Fig. 2:**
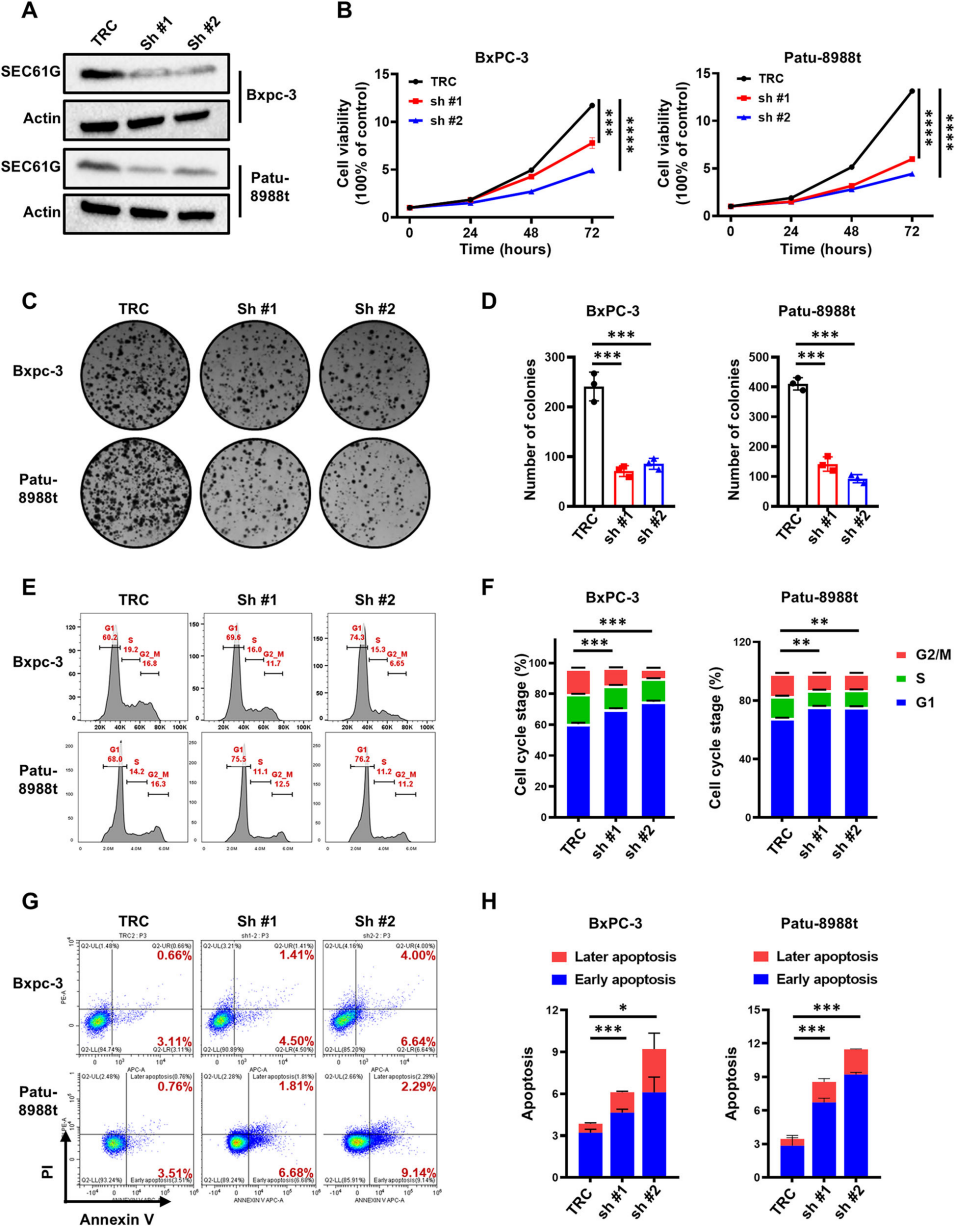
SEC61G promotes PDAC cell proliferation. **A** SEC61G expression was detected in the indicated cell lines by Western blot. **B** Cell growth curves of BxPC-3 and Patu-8988 T cell lines with wild or downregulated expression of SEC61G via CCK8 assay. The cell viability at the 72nd hour was compared between control cells and SEC61G-downregulated cells using an unpaired t-test. Colony-forming assay was performed in BxPC-3 and Patu-8988t cells with wild or downregulated expression of SEC61G, (**C**) for representative figures and **D** for statistical analysis between indicated cells. unpaired t test. Cell cycle analysis of indicated BxPC-3 and Patu-8988t cells, representative data was shown in (**E**) and statistical results in (**F**). unpaired t test. Apoptosis analysis of BxPC-3 and Patu-8988t cells with distinct expression of SEC61G, representative figures were shown in (**G**) and later apoptosis fraction of each group was compared using unpaired t test in (**H**). NS, no significance, *p < 0.05, **p < 0.01, ***p < 0.001.

To explore the biological function of SEC61G in PDAC, we first measured its expression across five PDAC cell lines (Fig. S2A) and then knocked down SEC61G using the pLKO.1 lentivirus system in two lines with distinct endogenous SEC61G expression levels (BxPC-3 and Patu-8988t) (Figs. 2A and S2B).

A series of in vitro proliferation assays, including CCK8, colony formation, cell cycle and apoptosis FACS test, were carried out. Findings showed that the cellular proliferation was largely halted when SEC61G was downregulated (Fig. 2B), which was in accordance with less colonies formulated in those groups (Fig. 2C, D). Moreover, the fraction of mitotic cells is evidently lower in the knockdown groups compared with the control group (Fig. 2E, F). At the same time, reduced expression of SEC61G resulted in a higher proportion of cells suffering apoptosis (Fig. 2G, H). As shown in Fig. S2C, D, additionally, migration ability was also blunted when SEC61G was reduced. We also measured the epithelial-mesenchymal transition (EMT) biomarkers in PDAC cell lines with different SEC61G expression. As shown in Fig. S2E, the EMT of PDAC cells was consistently reduced when SEC61G was knocked down in both cell lines. To summarize, SEC61G positively contributed to tumor malignant behaviors in vitro, including proliferation and migration.

### SEC61G accelerates tumor growth when implanted subcutaneously

To further confirm the pro-tumor role of SEC61G in PDAC growth in vivo, we subcutaneously injected PDAC cells (Patu-8988t cells endogenously expressing vector or shRNA mentioned above) in NCG mice. Tumor growth was monitored every five days until the 30^th^ day after implantation, when tumor-bearing mice were sacrificed, and tumors were removed. Additionally, those samples were subject to immunohistochemistry for cell proliferation assay as well as TUNEL staining for apoptosis detection in vivo.

**Fig. 3:**
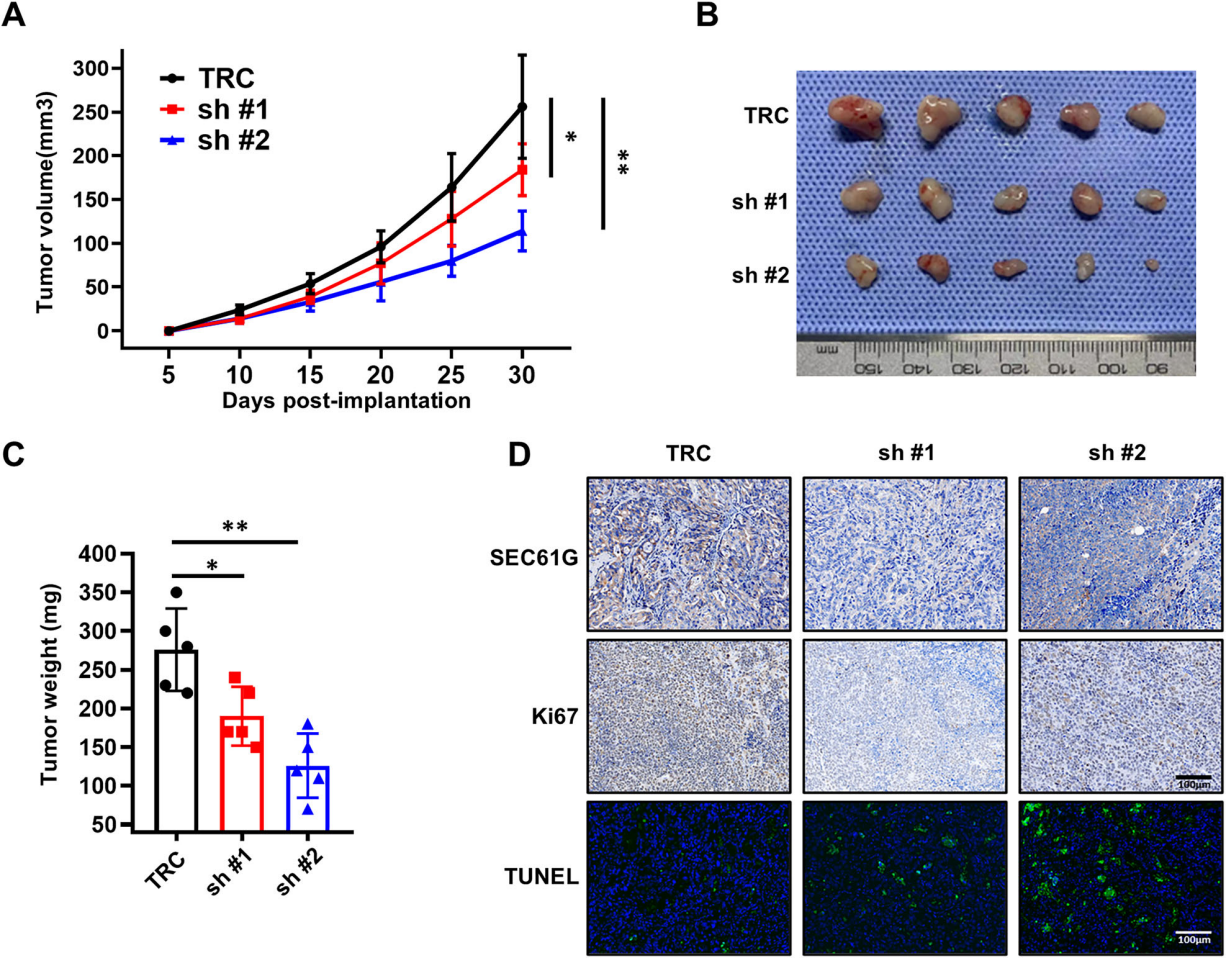
Tumor growth is promoted by SEC61G in vivo. **A** Growth curves of Patu-8988t cells with distinct expression of SEC61G inoculated subcutaneously in NCG mice. unpaired t test for tumor volume at the 30^th^ day. The volumes at the 30^th^ day after implantation were compared between the control tumors with indicated SEC61G-downregulated tumors using unpaired t test. **B** Representative figure of tumors in (**A**). **C** Tumor weight was analyzed by unpaired t test. **D** Represent immunohistochemical (SEC61G and Ki67) as well as TUNEL results for tumors, scale bar shown is 100μm. *p < 0.05, **p < 0.01.

Tumor growth was markedly halted in the SEC61G knockdown group (Fig. 3A). Both tumor volume and weight were significantly reduced in mice bearing SEC61G-silenced tumors (Fig. 3B, C). Moreover, the level of Ki67, reflecting cellular proliferation in vivo, was markedly decreased in these tumors (Fig. 3D). Additionally, the TUNEL staining showed that cell apoptosis was evidently enhanced in tumors with lower expression of SEC61G (Fig.3D), which was consistent with our in vitro results. These results confirm that SEC61G enhances PDAC tumor growth in vivo.

### SEC61G regulates autophagy in PDAC

**Fig. 4:**
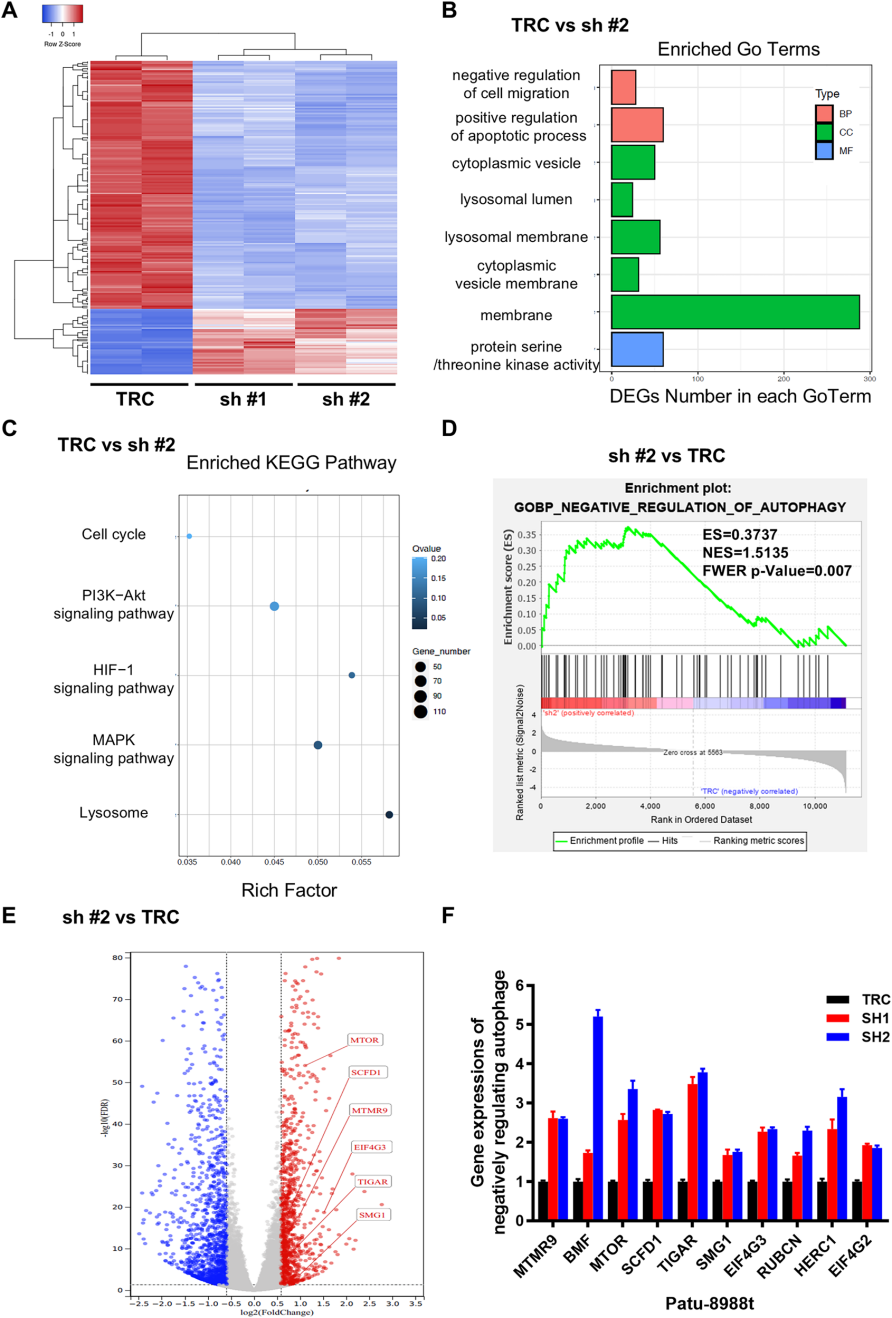
sec61g is associated with autophagy in Patu-8988t PDAC cells. **A** Heatmap showing gene expression in indicated Patu-8988t cell with different expression of sec61g. **B** Gene ontology (GO) presenting biological terms with significant differences between indicated cells. **C** Kyoto Encyclopedia of Genes and Genomes (KEGG) pathway analyzing signals with significant differences for indicated cells. |log2 (fold change) | > 1, *P* < 0.05. **D** Gene set enrichment analysis (GSEA) manifesting the different expression of genes among the pathways for negatively-regulating autophagy. **E** Volcano plots indicating the representative genes related in fig. (**D**). cutoff value: |log2 (fold change) | > 1, -log10(FDR) ≥ 2. (**F**). Expression of indicated genes was tested by RT-qPCR.

**Fig. 5:**
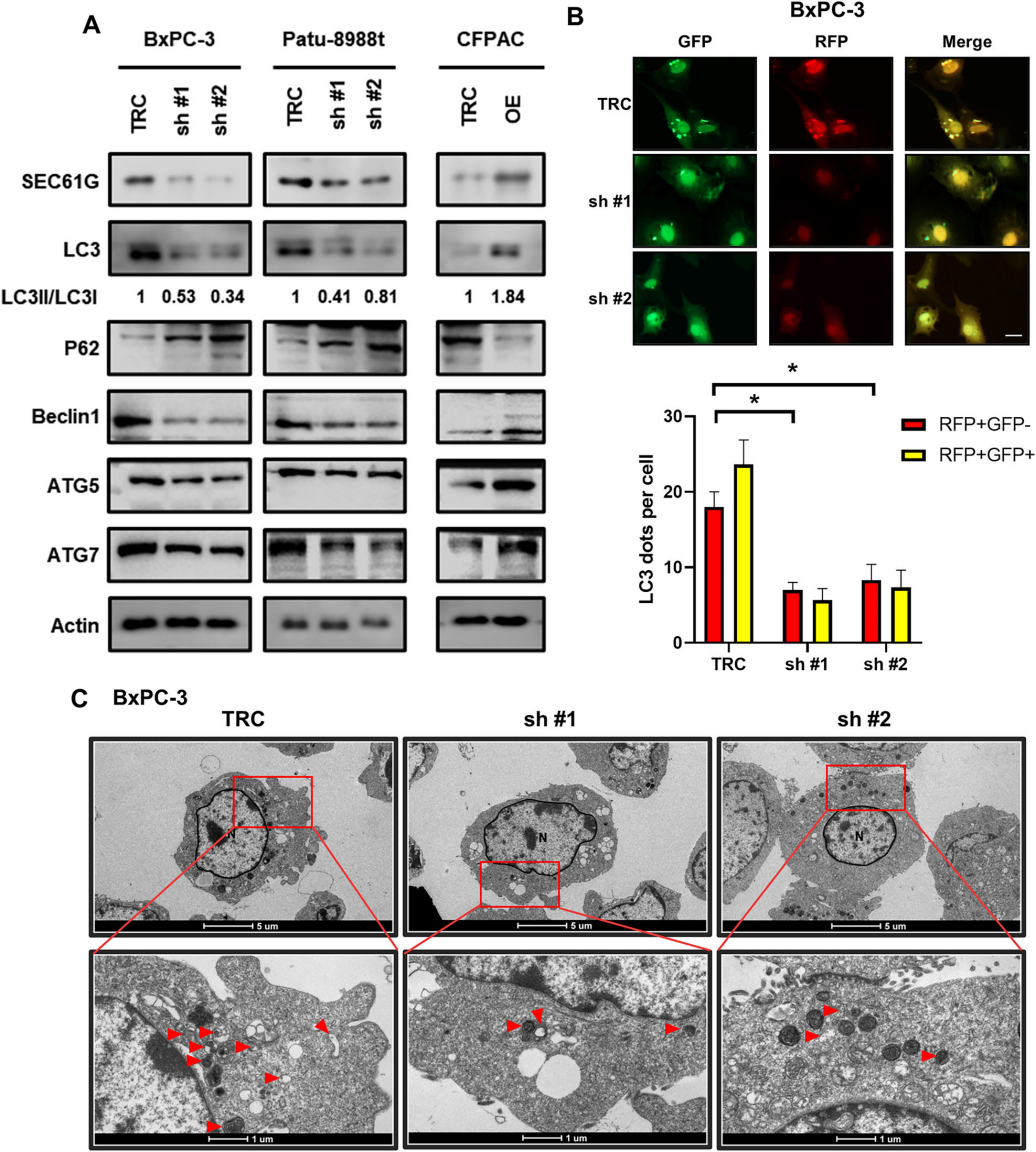
sec61g is associated with autophagy in Patu-8988t PDAC cells. **A** Expression of indicated protein was detected in different cell lines by Western blot. **B** Fluorescence photography of Patu-8988 T cell lines expressing mCherry-GFP-LC3 for autophagy flux detection. Red puncta (RFP^+^GFP^-^) signify autolysosomes and yellow (RFP^+^GFP^+^) puncta signify autophagosomes, scale bar shown is 10 μm (upper panel); LC3 puncta were quantified and analyzed using unpaired t test (bottom panel), *p < 0.05. **C** Transmission electron microscope analyzing the formulation of autophagosome in 591 indicated cells.

To unveil the mechanism underlying the SEC61G-mediated pro-tumor effect, RNA-sequencing was performed to determine the transcriptional differences between Patu-8988t cells with reduced SEC61G expression or not (Figs. 4A and S3A). Meanwhile, Gene Ontology (GO) analysis revealed widespread transcriptional alterations upon SEC61G silencing (Fig. 4B). In-depth analysis with the Kyoto Encyclopedia of Genes and Genomes (KEGG) (Fig. 4C) and GSEA analysis (Figs. 4D and S3B) demonstrated that SEC61G was involved in the regulation of a large number of pathways (like PI3K-Akt, HIF-1alpha and MAPK pathways) as well as cellular functions, especially autophagy, in a negative manner. As illustrated in the Volcano plots (Figs. 4E and S3C), knockdown of SEC61G increased the expression of autophagy-associated genes (like MTMR9, BMF) that were involved in the negative regulation of autophagy. These transcriptomic changes were further validated by RT-qPCR in Patu-8988t and BxPC-3 cells (Figs. 5F and S3D). Together, these findings suggest that SEC61G is involved in regulating autophagy in PDAC.

### SEC61G enhances autophagy in PDAC cells

It has been demonstrated that autophagy plays an essential role in maintaining cellular homeostasis, which is “hijacked” by tumor cells to overcome extracellular and intracellular stresses, especially in PDAC cells [9].

To determine whether SEC61G modulates PDAC autophagy, autophagy-related markers, including LC3, P62, Beclin 1, ATG5 and ATG7, were determined by western blotting. As shown in Figs. 5A and S4A, autophagy levels (reflected by the ratio of LC3-II to LC3-I as well as the expression of Beclin 1, ATG5 and ATG7) were evidently lower when SEC61G was knocked down in BxPC-3 and Patu-8988t cells, whereas those proteins were increased in CFPAC cells with overexpression manipulation. The expression of P62, acting as “fuel” in the autophagy process, changed in an opposite trend to that of LC3-II, implying that SEC61G is dispensable for autophagy flux. Furthermore, we also carried out transmission electron microscopy (TEM) to observe cellular autophagosomes and confirmed that the formation of autophagosomes was largely impaired following SEC61G knockdown in BxPC-3 cells (Fig. 5C). Consistently, more autophagosomes were detected in the CFPAC cells with overexpression of SEC61G compared with the counterpart (Fig. S4C).

As autophagy flux is a dynamic process, the increased number of autophagosome observed in TEM may result from enhanced autophagosome formulation or halted clearance of autophagosome. To address this, we first blocked the autophagy flux using chloroquine (CQ) and activated it with rapamycin (RAP). As shown in Fig. S4D, both CQ and RAP increased the expression of LC3II. P62, however, was increased in CQ-treated cells but decreased in RAP-treated cells. Overexpression of SEC61G shared the same trend with RAP in the changes of both LC3II and P62 accumulation, indicating that SEC61G, similar to rapamycin, may activate autophagosome formation in PDAC autophagy. To further assess autophagic flux, the mCherry-GFP-LC3 adenovirus was used to infect PDAC cell lines. The GFP signal is sensitive to pH and quenched in the lysosome for the acidic environment, whereas mCherry is stable in the lysosome. Therefore, the fusion of autophagosomes with lysosomes results in mCherry^+^GFP^-^ (red) puncta. As shown in Figs. 5B and S4E, mCherry^+^GFP^-^ puncta were reduced in PDAC cells with lower expression of SEC61G. However, the percentages of mCherry^+^GFP^+^ puncta were comparable between cells with different SEC61G expression (Fig. S4B, E). These autophagy flux results indicated that SEC61G promoted autophagy in PDAC, and the autophagy flux remained unimpeded during SEC61G manipulation. According to these findings, we presume that SEC61G activates autophagosome formation and therefore promotes PDAC proliferation.

To unveil the relationship between SEC61G and autophagy in patients with PDAC, we used online databases (TCGA and GEO) as well as 16 PDAC tumor specimens to examine the correlation between the expression of SEC61G and LC3B. The results showed a positive correlation at both the transcriptional (Fig. S5A) and protein (Fig. S5B) levels, indicating that SEC61G plays a promoting role in PDAC tumor autophagy.

### CREB enhances SEC61G-mediated Ca^2+^ leakage and therefore autophagy

**Fig. 6:**
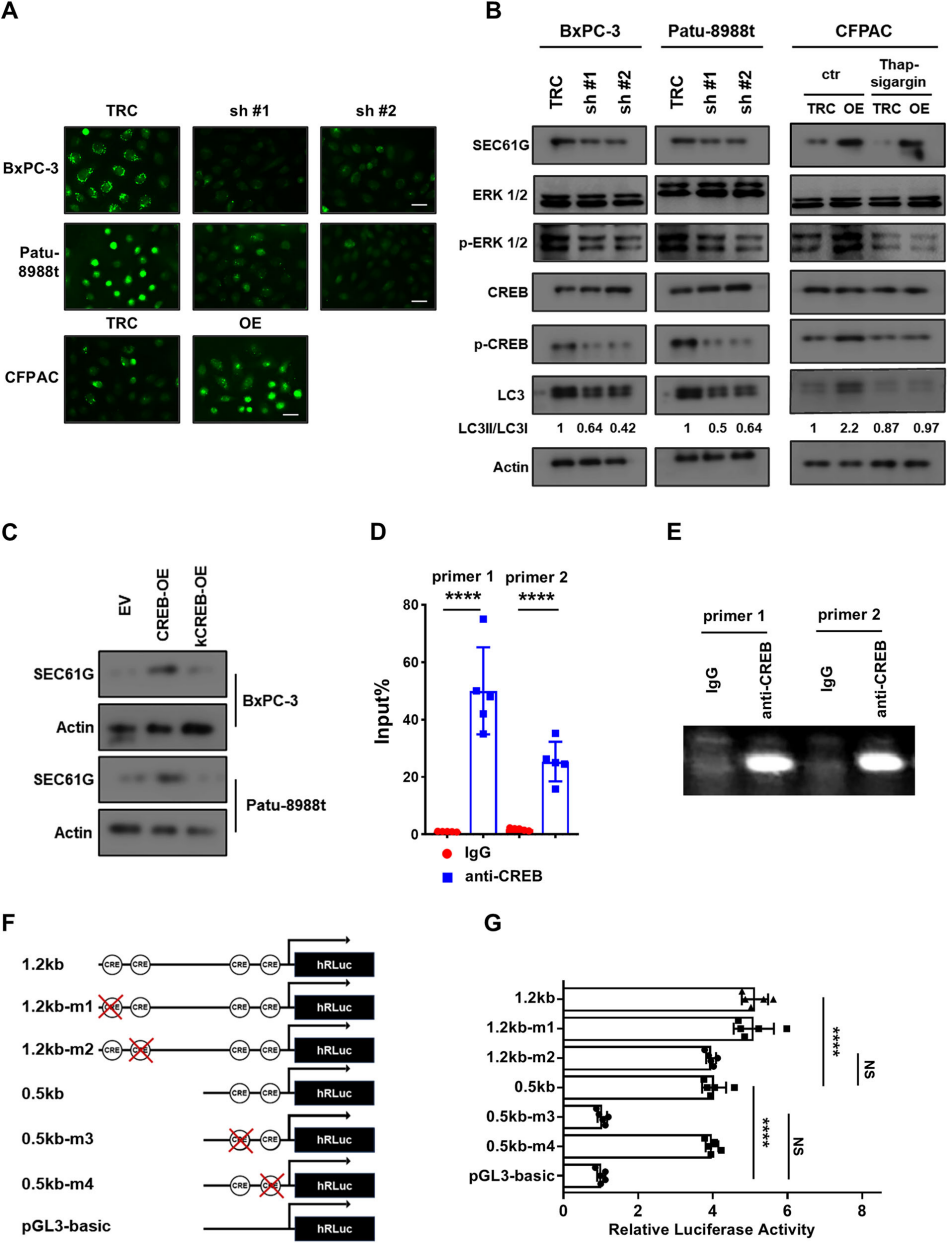
CREB-SEC61G loop is involved in calcium release from the endoplasmic reticulum. **A** Calcium in cytoplasm was presented through fluorescence staining, scale bar shown is 20μm. **B** Expression of indicated protein was detected by western blotting. **C** SEC61G expression was determined in indicated Patu-8988t cells expressing CREB with function or not. **D** CHIP assay indicating that CREB might enrich in promoter of sec61g with two distinct regions amplified by indicated primers. unpaired t test. **E** Amplified nucleic acids was presented by electrophoresis. **F** Schema for construction of plasmids with different length of sec61g promoter, with mutation or not, which were utilized in dual-luciferase assay present in (**G**). Dual-luciferase reporter experiment verified the facilitating effect of CREB in sec61g promoter. NS, no significance, ****p < 0.0001.

The SEC61 complex facilitates Ca²⁺ leakage from endoplasmic reticulum (ER) into cytosol [12], which can activate downstream Ca²⁺-dependent signaling pathways. It has been recently documented that SEC61G promotes stemness in head and neck squamous cell carcinoma via a Ca^2+^-dependent manner [22]. We therefore examined whether SEC61G promotes autophagy in PDAC through a similar mechanism, and detected the cellular Ca^2+^ concentrations in PDAC cell lines with different SEC61G expression levels. As shown in Fig. 6A, the intracellular level of Ca^2+^ was significantly reduced when SEC61G was knocked down in BxPC-3 and Patu-8988t cells, but was elevated in SEC61G-overexpressing CFPAC cells.

Correspondingly, the phosphorylation of ERK1/2 and CREB signaling pathways, which have been demonstrated to be activated by Ca^2+^ and involved in autophagy [8], was also diminished by SEC61G knockdown in both BxPC-3 and Patu-8988t cells (Fig. 6B, left panel). Thapsigargin, an ER Ca²⁺ release inhibitor, indeed reversed SEC61G-induced p-ERK1/2 and p-CREB activation (Fig. 6B, right panel). Intriguingly, SEC61G was downregulated in wild-type CFPAC cells treated with thapsigargin (Fig. 6B right panel, lane 1 vs lane 3), indicating that intracellular Ca²⁺ might be involved in regulating SEC61G expression.

CREB, a Ca^2+^-sensitive transcription factor, could transport calcium signaling from the cytoplasm into the nucleus. We speculated that the downregulation of SEC61G in thapsigargin-treated PDAC cells might be mediated by CREB. To verify our speculation, we first overexpressed CREB and kCREB, a dominant-negative CREB, in BxPC-3 and Patu-8988t cells. Our results showed that CREB indeed increased the expression of SEC61G, whereas kCREB suppressed it (Fig. 6C). Next, we analyzed the binding domains of CREB within the sec61g promoter using the online JASPAR database (https://jaspar.elixir.no/) and identified four putative CREB-binding sites within two regions of the sec61g promoter (Fig. S6C). ChIP assay was carried out using two pairs of primers targeting the binding regions mentioned above, and the results showed that CREB could bind to both regions (Fig. 6D, E). Lastly, a dual-luciferase assay system was designed using pGL3 system, within which promoters of sec61g (with mutation of potential binding points or not) was infused before Luciferase gene (Fig. 6F). Consist with the ChIP assay, the full length of sec61g promoter (−1.2bk ~ 200b) could activate full luciferase activity compared with the truncated promoter (−0.5 kb~200b), which was reduced only by mutation at binding sites 2 (Fig. 6G). Meanwhile, mutation at binding sites 3 also decreased luciferase activity in the truncated promoter (−0.5 kb~200b), which indicated that the binding sites 2 and 3 might be the targets combined by CREB.

To further demonstrate the causal relationship between SEC61G and CREB in PDAC autophagy, BxPC-3 cells with CREB overexpression were infected with shRNA targeting SEC61G or not (Fig. S6B, left panel). Cellular autophagy was activated when CREB was overexpressed, which was reversed by the knockdown of SEC61G. These findings indicated that SEC61G is indispensable for CREB-mediated activation of autophagy in PDAC cells. On the other hand, kCREB overexpression showed little effect on the elevation of autophagy induced by SEC61G overexpression (Fig. S6B, right panel). These results implied the pivotal role of SEC61G in regulating PDAC autophagy, which could not be blocked by the deletion of CREB. Collectively, CREB directly binds to the sec61g promoter and therefore facilitates its transcription, which in turn leads to Ca²⁺ leakage from ER and hence reinforces CREB activity. Therefore, the CREB-SEC61G positive feedback loop might be responsible for the highly activated status of autophagy in PDAC, in which SEC61G acts as both a “starter” and an “accelerator” to fuel cellular autophagy.

### Blocking ER Ca^2+^ leakage reverses SEC61G-mediated pro-tumor efficacy

**Fig. 7:**
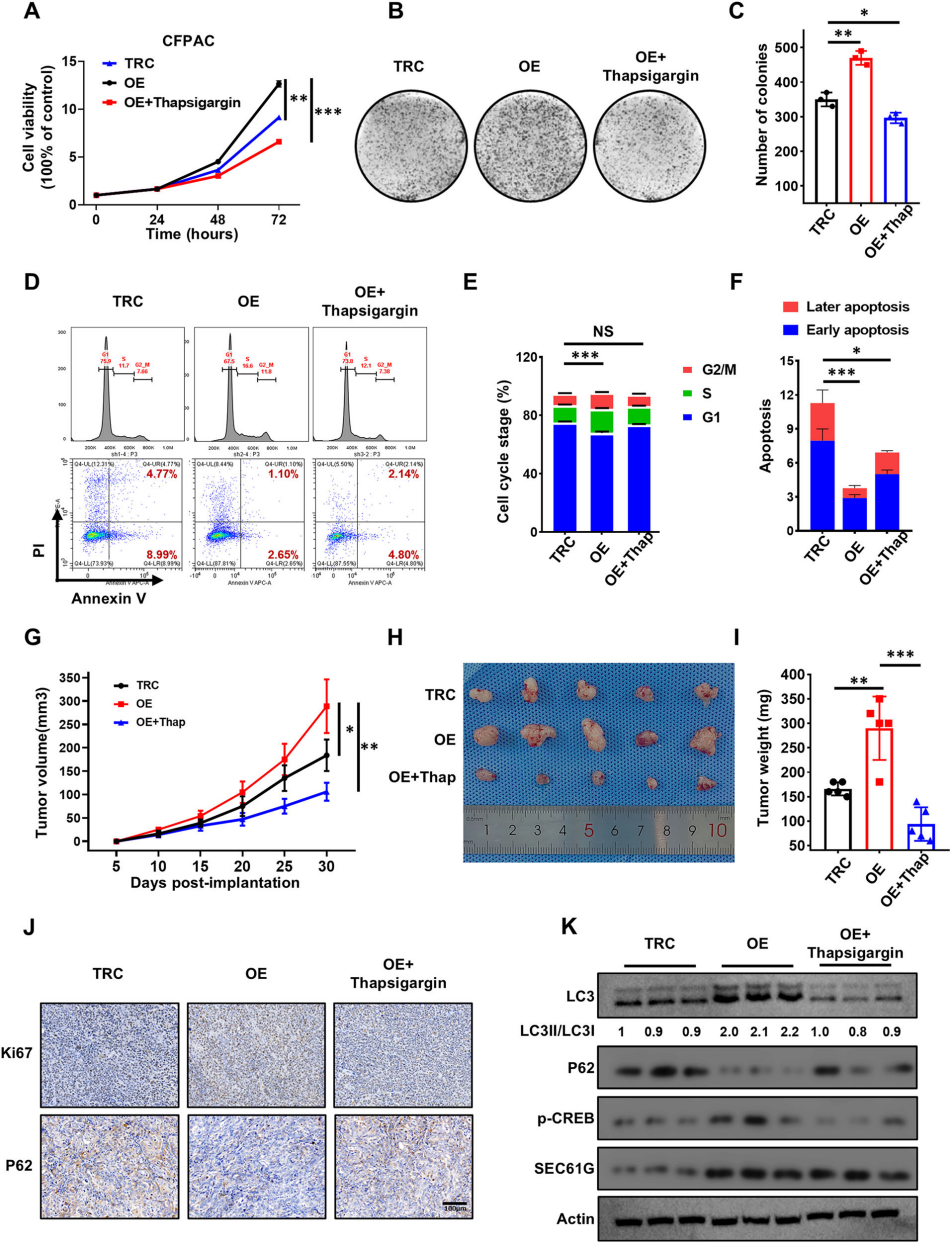
Blockade of calcium release from the ER hinders the pro-tumor effect of SEC61G. **A** Cell growth curve of CFAPC cells with wild or upregulated expression of SEC61G via CCK8 assay. The cell viability at the 72^nd^ hour between CFPAC cells with SEC61G overexpression or not was compared. Additionally, the cell viability between SEC61G-overexpressing CFPAC cells with thapsigargin treatment or not was also compared. Unpaired t test. **B**, **C** Colony-forming assay was performed in CFAPC cells with wild or upregulated expression of SEC61G, **B** for representative figures and **C** for statistical analysis between indicated cells, unpaired t test. (**D** up panel, **E**). Cell cycle analysis of indicated CFPAC cells, representative data was shown in **D** up panel and statistical results in **E**, unpaired t test. **D** bottom panel, **F**. Apoptosis analysis of CFPAC cells with distinct expression of SEC61G, representative figures were shown in D bottom panel and later apoptosis fraction of each group was compared using unpaired t test in (*F*). **G** Growth curves of CFPAC cells with distinct expression of SEC61G inoculated subcutaneously in NCG mice treated with thapsigargin or not. The volumes at the 30^th^ day after implantation were compared between tumors with SEC61G overexpression or not was compared. We also compared the volume between SEC61G-overexpressing tumors treated with thapsigargin or not. Unpaired t test. **H** Representative figure of tumors in (**G**). **I** Tumor weight was analyzed by unpaired t test. (**J**). Represent immunohistochemical results of tumors for indicated proteins, scale bar shown is 100μm. **K** Tumor expression of indicated protein detected with western blot. NS, no significance, *p < 0.05, **p < 0.01, ***p < 0.001.

To confirm that SEC61G promotes PDAC progression via ER Ca²⁺ leakage, SEC61G-overexpressing CFPAC cells (Fig. S6D) were treated with thapsigargin to deplete ER Ca²⁺ release. As expected, thapsigargin treatment largely abolished the pro-proliferative effects of SEC61G in vitro, as shown by CCK-8, colony formation, cell cycle and apoptosis assays (Fig. 7A–F). Similarly, thapsigargin administration also suppressed the accelerated tumor growth observed in SEC61G-overexpressing xenografts (Fig. 7G–I). The IHC (Fig. 7J and S6E) and western blot (Fig. 7K) results further indicated that thapsigargin inhibited autophagy in PDAC tumors in vivo, as reflected by the decreased LC3B and increased P62 expression. Taken together, these results demonstrate that SEC61G promotes PDAC progression both in vitro and in vivo in a Ca²⁺ leakage-dependent manner.

## Discussion

SEC61G is a core subunit of the SEC61 complex, which plays an essential role in protein maturation and translocation across the endoplasmic reticulum (ER) membrane. Recently, accumulating evidence has indicated that SEC61G contributes to tumorigenesis through multiple mechanisms. In glioblastoma, for example, SEC61G has been reported [13] to be co-amplified with EGFR at the 7p11 locus, resulting in its overexpression. Elevated SEC61G expression enhances the presentation of immune checkpoint ligands (ICLs) on the cell membrane, thereby facilitating tumor immune evasion and glioblastoma progression. In breast [15] and lung [23] cancer, SEC61G, however, was reported to modulate cellular glycolysis and therefore enable tumor cells to adapt to nutrient deprivation. In addition, SEC61G was also illustrated to facilitate tumor progression by promoting Ca^2+^ leakage from ER into cytosol [17]. Although previous studies have suggested that SEC61G promoted tumor growth in pancreatic ductal adenocarcinoma (PDAC), the underlying molecular mechanism remained largely undefined [24]. In our study, we demonstrated that SEC61G is highly expressed in PDAC tissues and correlates with poor overall survival in patients (Fig. 1 and S1), implying that SEC61G might act as a potential biomarker to predict PDAC prognosis. Furthermore, both in vivo and in vitro experiments were carried out to confirm the pro-tumorigenesis effects of SEC61G in PDAC (Figs. 2, 3). Mechanistically, SEC61G enhanced Ca^2+^ leakage from ER and subsequently fueled cellular autophagy in PDAC cells (Figs. 4, 5). Notably, this effect was effectively abolished by thapsigargin, a specific inhibitor of ER Ca²⁺ release (Fig. 7).

Autophagy plays a dual role in cancer, functioning as both a tumor-suppressive and a tumor-promoting process depending on the context [25, 26]. During tumor initiation, autophagy can prevent genomic instability, promote apoptosis following chemotherapy and clear damaged organelles, which could otherwise drive oncogenic transformation [27, 28]. On the other hand, autophagy sustains cancer cell metabolism and survival under stress, as well as suppresses immune attack in the progression of the tumor [29]. It is well documented that autophagy is prevalently activated in PDAC [10], and targeting autophagy might be a potential strategy for fighting PDAC progression [7].

**Fig. 8:**
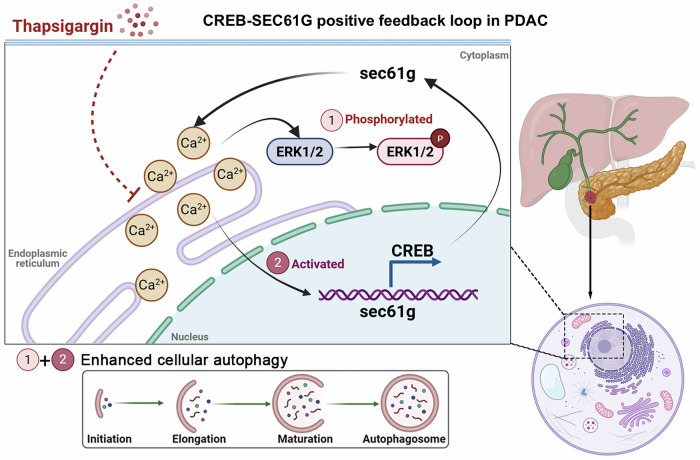
Schematic illustration showing the mechanism of CREB-SEC61G positive feedback loop in fueling cellular autophagy in PDAC. Elevated SEC61G releases Ca^2+^ stored in endoplasmic reticulum into cytosol and therefore activates the phosphorylation of CREB. In turn, activated p-CREB binds with the promoter of sec61g and hence promote the expression of SEC61G, which forms the CREB-SEC61G positive feedback loop in PDAC. At the same time, increased concentration of Ca^2+^ by SEC61G overexpression also activates autophagy-related pathways, like ERK1/2, and sustains PDAC survival in harsh environment.

In the present study, we further found that the high expression of SEC61G was attributed to the activation of CREB, which could bind to the promoter and therefore enhance the transcription of sec61g (Fig. 6). Intriguingly, Ca^2+^ leakage from ER by SEC61G led to phosphorylation and activation of CREB, which was verified both in vitro and in vivo (Figs. 6B and 7K). While CREB-mediated transcription downstream of Ca²⁺ signaling was well known, the downstream effectors that connect CREB activity back to ER Ca²⁺ dynamics remain poorly defined. Our study established SEC61G as a previously unidentified effector linking CREB activation to ER Ca²⁺ homeostasis. The positive feedback loop formed by CREB and SEC61G (Fig. 8) might answer the question why autophagy was persistently activated in PDAC, and, as a result, downregulation of SEC61G expression or blockade the Ca^2+^ released from ER was the key to quench the highly activated status of autophagy in PDAC. Also, our findings placed CREB within a previously uncharacterized regulatory circuit that functionally links ER Ca²⁺ dynamics to autophagy-dependent tumor progression.

Although we did not directly target SEC61G using pharmacological inhibitors, due to the current lack of specific agents, our study provided functional evidence supporting its therapeutic potential. Pharmacological inhibition of ER Ca²⁺ signaling with thapsigargin significantly abrogated SEC61G-mediated tumor growth both in vitro and in vivo (Fig. 7). We acknowledge, however, that thapsigargin is a broad inhibitor of ER Ca²⁺ signaling and therefore may induce systemic side effects when applied in tumor treatment, including PDAC. Consequently, further efforts are warranted to develop tumor-targeted or pancreas-specific inhibitors against SEC61G or ER Ca²⁺ signaling for future PDAC therapy.

Taken together, our study unveiled that SEC61G was a key driver in PDAC progression and also a potential indicator for patient prognosis. Moreover, we uncover a novel mechanism, the CREB-SEC61G positive feedback loop, underlying persistent autophagy activation in PDAC. Furthermore, we provide new mechanistic insights as well as a potential therapeutic candidate for combating this highly lethal malignancy.

## Conclusion

Our research indicates that SEC61G acts as an oncogene in PDAC and highlights the essential role of the CREB-SEC61G feedback loop in sustaining the activation of autophagy in PDAC. These findings contribute to predicting patient prognosis based on SEC61G expression and propose a strategy of targeting SEC61G as a potential therapy to quench autophagy for PDAC treatment, which requires further validation.

## Supplementary information

Supplementary information

supplementary Figure 1

supplementary Figure 2

supplementary Figure 3

supplementary Figure 4

supplementary Figure 5

supplementary Figure 6

uncropped original western blots

## Data Availability

All data responsible for evaluating the conclusions in the paper are presented in the paper and/or the Supplementary Materials. The datasets used and analyzed during the study are available from the corresponding author on reasonable request.
