# Supplementary material for: CREB-SEC61G feedback loop sustains enhanced autophagy and boosts proliferation in PDAC
Source: Cell Death Dis. 2026 May 29;17(1):669. doi: 10.1038/s41419-026-08915-7 (PMC13424619; doi:10.1038/s41419-026-08915-7)
Supplement: Supplementary file 1 — Supplementary information [file 41419_2026_8915_MOESM1_ESM.docx]

**Supplementary information for**

**CREB-SEC61G feedback loop sustains enhanced autophagy and boosts proliferation in PDAC**

Xiao Wu et al.

This file contains following information:

Supplementary tables 1-6；

Supplementary methods;

Supplementary figure legends: Figure S1-6.

**Supplementary tables**

**Supplementary table 1. The clinically pathological characteristics of 70 PDAC patients.**

| Characteristics | Values |
| --- | --- |
| Age, year (mean ± SD) | 62.79 ±10.74 (34-82) |
| Gender (male/female) | 33/37 |
| CA19-9, U/ml (≤37/＞37) | 15/55 |
| TNM stage (I/II/III/IV)  Lymphatic metastasis (Negative, Positive) | 27/18/19/6  38/32 |
| Cancer embolus (Negative, Positive) | 41/29 |
| Vascular invasion (Negative, Positive) | 53/17 |
| Perineural invasion (Negative, Positive) | 48/22 |
| SEC61G IHC score (＜4/≥4) | 33/37 |
| Overall survival, months (mean ± SD) | 14.63 ± 11.11 (2-51) |

**Table 2. Prognostic factor for OS of patients with PDAC determined by using univariate and multivariate.**

| Variable | | | | Overall survival | | | | | |
| --- | --- | --- | --- | --- | --- | --- | --- | --- | --- |
|  |  |  |  | HR | | 95%CI | | *P*-value | |
| **Univariate** | | | |  |  |  |  |  |  |
| Age(＜62 or≥62) | | | | 1.159 | | 0.704 - 1.910 | | 0.5451 | |
| Gender(female/male) | | | | 1.132 | | 0.673 - 1.902 | | 0.6405 | |
| CA19-9(＞37U/ml or ≤37U/ml) | | | | 2.120 | | 1.240 - 3.625 | | 0.0151 | |
| TNM stage (I+II/III+IV) | | | | 1.861 | | 1.050 - 3.298 | | 0.0113 | |
| Lymphatic metastasis (+/-) | | | | 2.450 | | 1.425 - 4.210 | | <0.0001 | |
| Cancer embolus (+/-) | | | | 0.652 | | 0.383 - 1.112 | | 0.0817 | |
| Vascular invasion (+/-) | | | | 1.693 | | 0.866 - 3.308 | | 0.1237 | |
| Perineural invasion (+/-) | | | | 1.207 | | 0.688 - 2.117 | | 0.4784 | |
| SEC61G protein level(＜4/≥4) | | | | 0.367 | | 0.217 - 0.621 | | <0.0001 | |
| **Multivariate** | | | |  |  |  |  |  |  |
| SEC61G protein level(＜4/≥4) | | | | 0.355 | | 0.206 – 0.612 | | 0.0001 | |
| TNM stage (I+II/III+IV) | | | | 1.908 | | 1.115 – 3.263 | | 0.018 | |
| Lymphatic metastasis (+/-) | | | | 2.391 | | 1.379 – 4.147 | | 0.002 | |

CA19-9 carbohydrate antigen 19-9; HR hazard ratio; CI confidence interval

According to Immunohistochemical (IHC) score, split at median.

**Table 3. Correlation between SEC61G expression and PDAC in 70 patient cohort.**

| Characteristics | Number of patients | | P-value^a^ |
| --- | --- | --- | --- |
|  | Low SEC61G expression | High SEC61G expression |  |
| **Gender** |  |  | **0.4551** |
| Female | 19 | 18 |  |
| Male | 14 | 19 |  |
| **Age** |  |  | **0.2413** |
| ＞62 | 18 | 15 |  |
| ≤62 | 15 | 22 |  |
| **Vascular invasion** |  |  | **0.0250** |
| Negative | 29 | 24 |  |
| Positive | 4 | 13 |  |
| **Cancer embolus** |  |  | **0.0232** |
| Negative | 24 | 17 |  |
| Positive | 9 | 20 |  |
| **Perineural invasion** |  |  | **0.4794** |
| Negative | 24 | 24 |  |
| Positive | 9 | 13 |  |
| **LN invasion** |  |  | **0.0496** |
| Negative | 22 | 16 |  |
| Positive | 11 | 21 |  |
| **TNM stage** |  |  | **0.0721^b^** |
| Ⅰ | 16 | 11 |  |
| Ⅱ | 8 | 10 |  |
| Ⅲ | 9 | 10 |  |
| Ⅳ | 0 | 6 |  |

**a. Chi-square test; b. Fisher's exact test**

**Supplementary table 4. The primers used for RT-qPCR.**

| genes | Forward primers | Reverse primers |
| --- | --- | --- |
| Sec61g | GCAGTTTGTTGAGCCAAGTCG | CCAGCCGAATGGAGTCCTT |
| mtmr9 | CCGGCAGGACAATACGGAG | ATGCCTCAATGGAACTGGCTA |
| bmf | CCAGAGCCTACTGGACTGC | AGCCAGCATTGCCATAAAAGA |
| mtor | TCCGAGAGATGAGTCAAGAGG | CACCTTCCACTCCTATGAGGC |
| scfd1 | ATGCAGCGTTAGCAGCTAGTG | GGCCTGTTAATGGCACGATATG |
| tigar | ACTCAAGACTTCGGGAAAGGA | CACGCATTTTCACCTGGTCC |
| smg1 | GGTGGCTCGATGTTACCCTC | CTGCGTGAGCGAAGGTTTC |
| Eif4g3 | CCTAGAGCTACCATCCCGAAC | GGGCCACTATGACGGTACTG |
| rubcn | CCTGCTCGGGGATAGACAGTA | CTGGCAAACATGGACGCATC |
| herc1 | AGCTGAAATGGCTTGAACACT | GCAACTCCCTCTCTTGTAGCA |
| Eif4g2 | GGGGTGCTTCTCGTTTCAGT | AGCAGTCTTGGGATAGTGCTG |
| gapdh | ACAACTTTGGTATCGTGGAAGG | GCCATCACGCCACAGTTTC |

**Supplementary table 5. Sequences of shRNA targeting indicated genes and CHIP primers.**

| genes | sequences |
| --- | --- |
| sec61g sh #1 | AGCCAAGUCGGCAGUUUGUAA |
| sec61g sh #2 | AUUCCAGAAGAUUGCCAUGGC |
| CHIP primer 1 forward | AGTAACCAGGAATTAGAGTTCTAGAG |
| CHIP primer 1 reversse | TACTCAGGAGGCTGAGGCA |
| CHIP primer 2 forward | CTTAGATGGTCACTATAGGCCT |
| CHIP primer 1 reversse | CAGAACCTCCAGATATTGATTCAGT |

**Supplementary table 6. Antibodies used in this paper.**

| **Antibodies** | **Sources** | **Identifiers** | **Application** |
| --- | --- | --- | --- |
| SEC61G | Protein tech | Cat# 17952-1-AP | IHC |
| SEC61G | Affinity | Cat# DF12136 | WB |
| Ki67 | Cell Signaling Technology | Cat# 9449 | IHC |
| LC3B | Cell Signaling Technology | Cat# 3868 | WB, IHC |
| P62 | Cell Signaling Technology | Cat# 88588 | WB, IHC |
| ERK 1/2 | ABclonal | Cat# A16686 | WB |
| P-ERK 1/2 | ABclonal | Cat# AP0974 | WB |
| CREB | Sigma Aldrich | Cat# 06-863 | WB, CHIP |
| p-CREB | Cell Signaling Technology | Cat# 9198 | WB |
| ACTIN | Cell Signaling Technology | Cat# 3700 | WB |
| Beclin 1 | Cell Signaling Technology | Ca# 3738 | WB |
| ATG5 | Cell Signaling Technology | Ca# 12994 | WB |
| ATG7 | Cell Signaling Technology | Ca# 2631 | WB |
| Tunel kit | Cell Signaling Technology | Ca# 25879 | IF |
| E-cadherin | Cell Signaling Technology | Ca# 3195 | WB |
| N-cadherin | Cell Signaling Technology | Ca# 13116 | WB |
| Vimentin | Cell Signaling Technology | Ca# 5741 | WB |
| Snail | Cell Signaling Technology | Ca# 3879 | WB |

**Supplementary materials and methods**

**Cell culture and transfection**

The human PDAC cell lines, BxPC-3, Patu-8988t and CFPAC, were purchased from Cellcook Cell Biotechnology (Guangzhou, China). The cells were cultured in DMEM basic media, supplemented with 10% fetal bovine serum (FBS; Gibco, #A3160802). Thapsigargin (0.1 μM) was used to treat cultured cells when needed. For SEC61G knockdown assays, PDAC cell lines, BxPC-3 and Patu-8988t, were transfected with lentivirus-encoding shRNAs using pLKO.1 system. The shRNA sequences used for silencing SEC61G and control (TRC) are provided in supplementary information.

**Western blot analysis**

Cell lysates were collected in RIPA lysis buffer (50mM Tris-HCl pH 7.4, 1mM EDTA, 0.25% deoxycholic acid disodium salt, 1% NP40, 150mM NaCl, 0.1% SDS) supplemented with protease inhibitor (TargetMol Chemicals, USA) and PMSF. After quantification by BCA assay, 30 μg of protein lysates were separated by SDS/PAGE and then transferred to a PVDF membrane (Bio-Rad, USA). After 1 h blockade, the membranes were incubated with appropriate antibodies overnight at 4 °C and then washed with TBST three times. Following incubation with secondary antibody conjugated with horseradish peroxidase for 1 h at room temperature, the protein-specific bands were detected using a chemiluminescence reagent (ECL, Millipore, USA). Experiments were repeated for at least three times.

**Real-time quantitative PCR**

Total RNA was extracted from cells using TRIzol reagent (Invitrogen) and then reversed transcribed with HiScript Q Select RT SuperMix (Vazyme Biotech) to synthesize cDNA. Quantitative real-time PCR was performed with SYBR Green Mix (Vazyme Biotech) and indicated primers were used to assess transcript level of each gene.

**Immunohistochemical staining**

IHC staining of paraffin-embedded tissues with indicated antibodies was performed according the previously described standard procedures. In Fig.1, The expression level of SEC61G was scored semi-quantitatively based on both intensity of staining and distribution using the immunoreactive score (IRS). Staining intensity (SI) was recorded as 0, 1, 2, 3, referring to negative, weak, moderate and strong staining respectively. Percentage of positive cells (PP) was assigned as 0=0%, 1=1-25%, 2=25-50%, 3=50-75%, 4=75-100%. Briefly, IRS = SI x PP (ranging from 1 to 12), and high expression referred to IRS more than 4.

**CCK8 assay**

Cell viability was measured at 0, 24, 48,72h for each cell line with Cell Counting Kit-8 (CCK8) (Dojindo, Japan) according to the manufacturer’s instructions. Absorbance values were measured from three repeated wells at the wavelength of 450 nm as representation of cell viability.

**clone formation assay**

Four hundred tumour cells were seeded per well into a six-well plate and cultured for 15 days. The conditioned medium was changed every 4 days. The cells were then fixed in 4% formaldehyde for 20 min and stained with 0.5% crystal violet for 30 min. Plates were washed twice using PBS, pictures were taken, and colony numbers were calculated using ImageJ from three different fields.

**Cell cycle assay**

The PDAC cells infected with different lentivirus. Cells were collected and then subjected to cell cycle assay using the cell cycle staining kit (BestBio, China). The data was collected by flow cytometry and analyzed with CytExpert software (Beckman Coulter). Representative images were shown in the paper and results from three independent experiments were collected for analysis.

**Apoptosis assay**

Percentages of apoptotic cell in infected cells were assessed using the Annexin V-FITC / PI Apoptosis Kit (BestBio, China) according to the manufacturer's instructions. The data was collected by flow cytometry and analyzed with CytExpert software (Beckman Coulter). Representative images were shown in the paper and results from three independent experiments were collected for analysis.

**Transwell invasion assays**

As previously described, 6×10^4^ cells suspended in 200 μL serum-free medium were seeded in the upper chamber coated with Matrigel (1:20 dilution; Corning, #354234). 24 hours later, invasive cells on the lower surface were fixed and then stained using 0.5% crystal violet for 15 min. The number of invaded cells was imaged and counted using a light microscope from three different fields for statistical analysis.

**Supplementary figure legends**

**Supplementary Fig 1.**

(A). Analysis of sec61a and sec61b expression in GSE16515 databases, unpaired t test.

(B). Analysis of sec61a and sec61b expression in paired GSE28735 databases, paired t test.

(C). Overall survival based on sec61g expression in GSE62452 database. Kaplan–Meier test, ns, no significance.

(D). sec61g expression in PDAC patients with distinct stages. Unpaired t test.

(E). Statistical analysis of IHC scores from TMA was performed, unpaired t test. ****p<0.0001.

**Supplementary Fig 2.**

(A). Transcription (upper) and protein (bottom) level of SEC61G was detected in different PDAC cell lines using RT-qPCR and western blot.

(B). Sec61g expression of indicated cells was determined via RT-qPCR. unpaired t test.

(C, D). Migration ability of indicated BxPC-3 and Patu-8988t cell lines with wild or downregulated expression of SEC61G detected by transwell assay. C for representative figures and D for statistical analysis between indicated cells, unpaired t test.

(E). Expression of EMT-associated proteins (including E-Cad, N-Cad, Vimentin, Snail) was detected in Patu-8988t and BxPC-3 cell lines with downregulation of SEC61G or not using western blot.

NS, no significance, *p<0.05, **p<0.01, ***p<0.001.

**Supplementary Fig 3.**

(A). Venny figures showing the common gene upregulated (up) and downregulated (bottom) between indicated cells.

(B). Gene set enrichment analysis (GSEA) manifesting the different expression of genes among the pathway for negatively-regulating autophagy.

(C). Volcano plots indicating the representative genes related in figure D. cutoff value: |log2 (fold change) | > 1, -log10(FDR) ≥2.

(D). Expression of indicated genes in BxPC-3 cells was tested by RT-qPCR.

**Supplementary Fig 4.**

(A). Ratios of LC3II to LC3I related to WB results shown in figure 5A were calculated and analyzed via unpaired t test.

(B). Percentages of GFP^+^RFP^+^ dots in indicated BxPC-3 cells expressing mCherry-GFP-LC3 related in figure 5B were compared using unpaired t test.

(C). Transmission electron microscope analyzing the formulation of autophagosome in CAPAC cells overexpressing SEC61G or not.

(D). Expression of autophagy protein markers (including LC3, p62, ATG5) was detected in Patu-8988t and BxPC-3 cell lines with overexpression of SEC61G or not, which were treated with autophagy agonist (Rapamycin, Rap) or autophagy inhibitor (chloroquine, CQ).

(E). Fluorescence photography of indicated Patu-8988t and CFPAC cells expressing mCherry-GFP-LC3 for autophagy flux detection. Red puncta (RFP^+^GFP^-^) signify autolysosomes and yellow (RFP^+^GFP^+^) puncta signify autophagosomes, scale bar shown is 10μm (Left panel); Quantification of indicated LC3 puncta (middle panel); Analysis of percentages of GFP^+^RFP^+^ dots in indicated cells (right panel). unpaired t test.

NS, no significance, *p<0.05, **p<0.01, ***p<0.001, ****p<0.0001.

**Supplementary Fig 5.**

(A). Correlation analysis between sec61g and lc3b expression based on online databases (TACG, GSE16515 and GSE28735).

(B). SEC61G and LC3 expression in 16 PDAC tumors were detected with western blot (upper panel); Correlation between the expression of SEC61G and LC3B were analyzed (bottom panel), linear-regression analysis.

**Supplementary Fig 6.**

(A). Ratio of p-ERK1/2 to ERK1/2 and p-CREB to CREB in figure 6B were calculated and analyzed using unpaired t test.

(B). To unveil the causal relationship between SEC61G and CREB in fueling autophagy, the expression of autophagy protein markers (including LC3, p62, Beclin 1, ATG5, ATG7) was detected via western blot in indicated BxPC-3 cell lines with manipulation of SEC61G and CREB expression.

(C). Possible binding points of CREB in sec61g promoter was shown in up panel and the detailed gene sequences in the bottom panel.

(D). SEC61G expression in CFPAC cell overexpressing SEC61G or not was determined via western blot.

(E). Represent immunohistochemical results of tumors for indicated proteins related in Fig.7J, scale bar shown is 100μm.

NS, no significance, **p<0.01, ***p<0.001, ****p<0.0001.
