# Supplementary figures and images for: CREB-SEC61G feedback loop sustains enhanced autophagy and boosts proliferation in PDAC
Source: Cell Death Dis. 2026 May 29;17(1):669. doi: 10.1038/s41419-026-08915-7 (PMC13424619; doi:10.1038/s41419-026-08915-7)

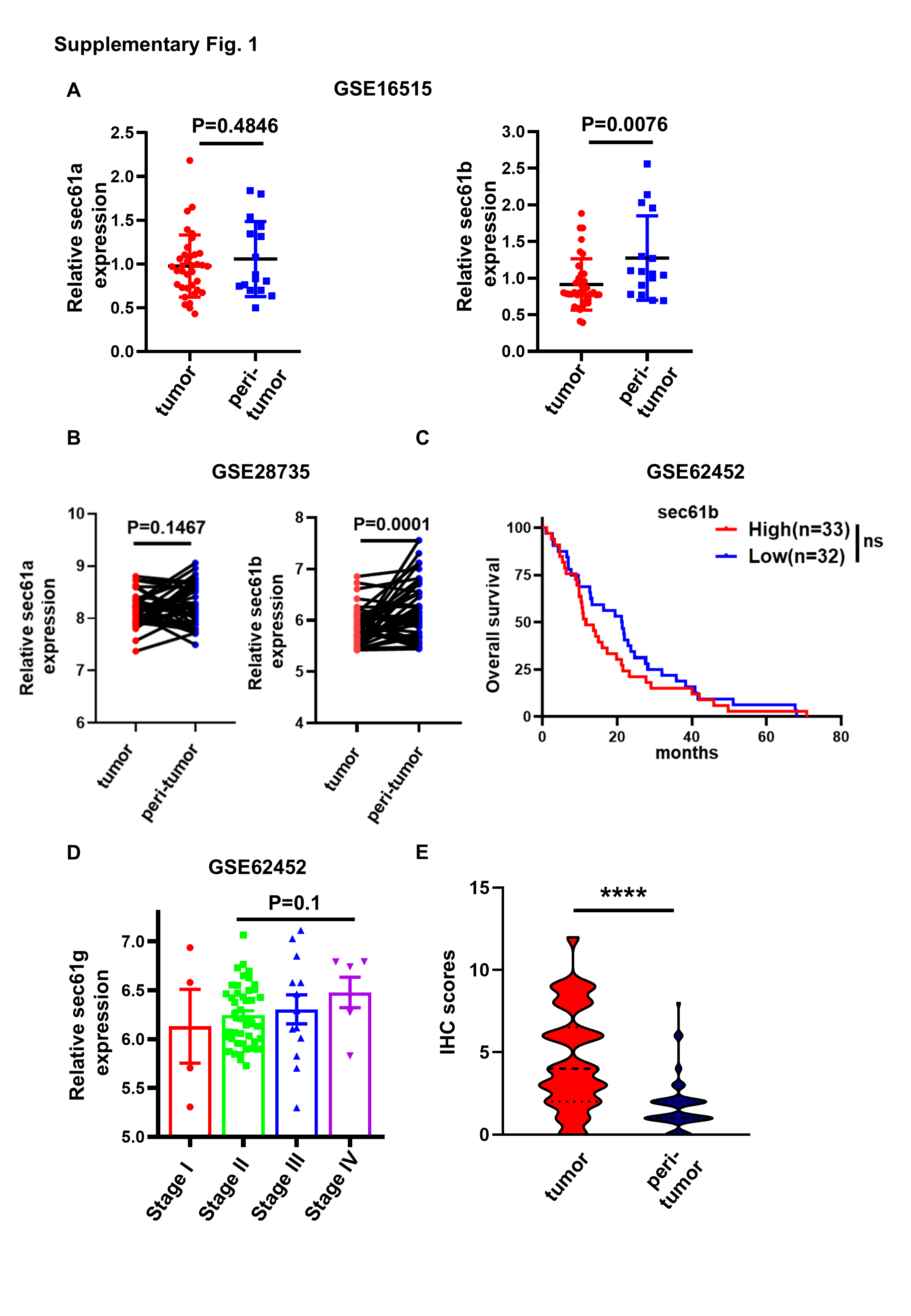

Supplement: Supplementary file 2 — supplementary Figure 1 [file 41419_2026_8915_MOESM2_ESM.tif]

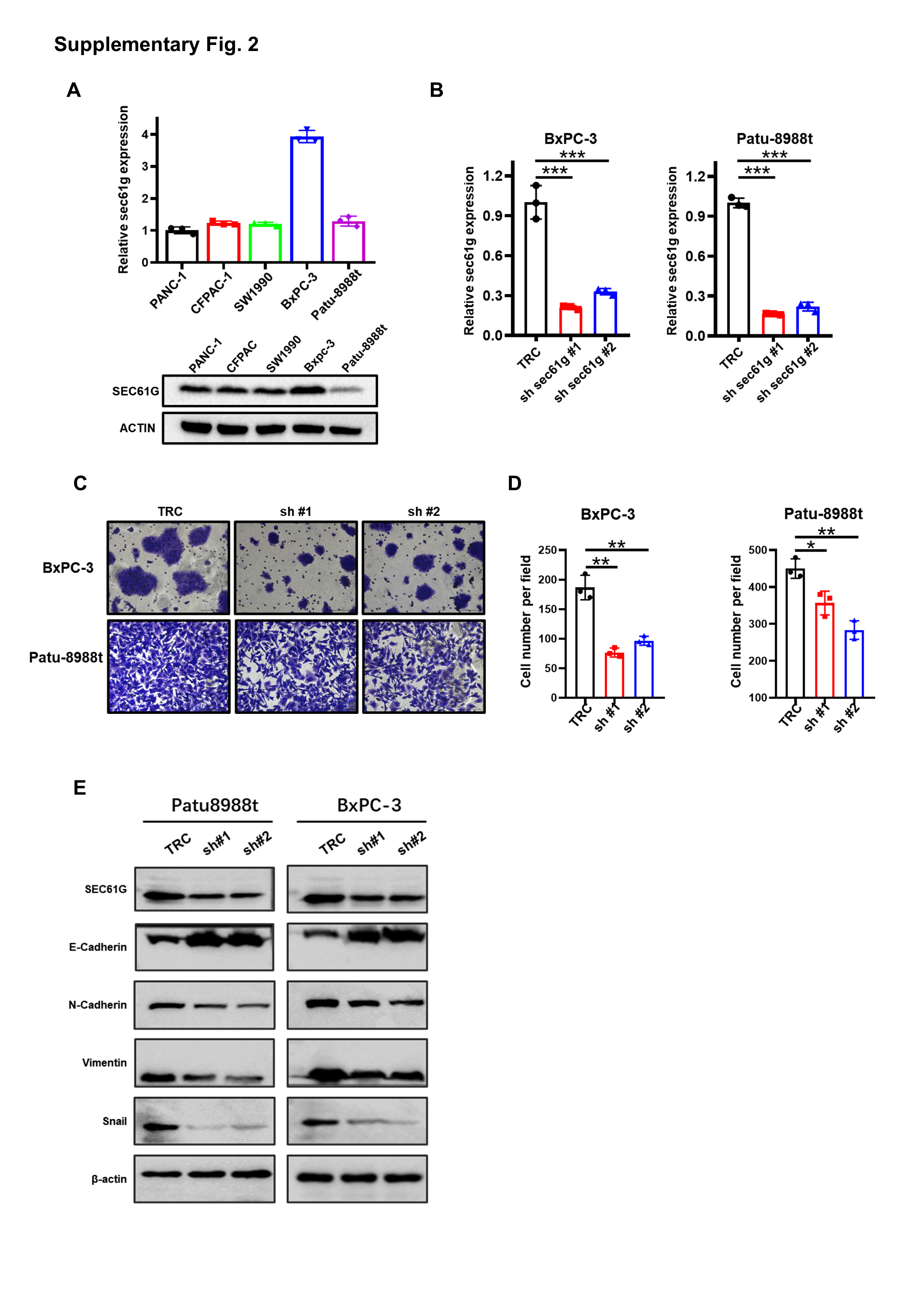

Supplement: Supplementary file 3 — supplementary Figure 2 [file 41419_2026_8915_MOESM3_ESM.tif]

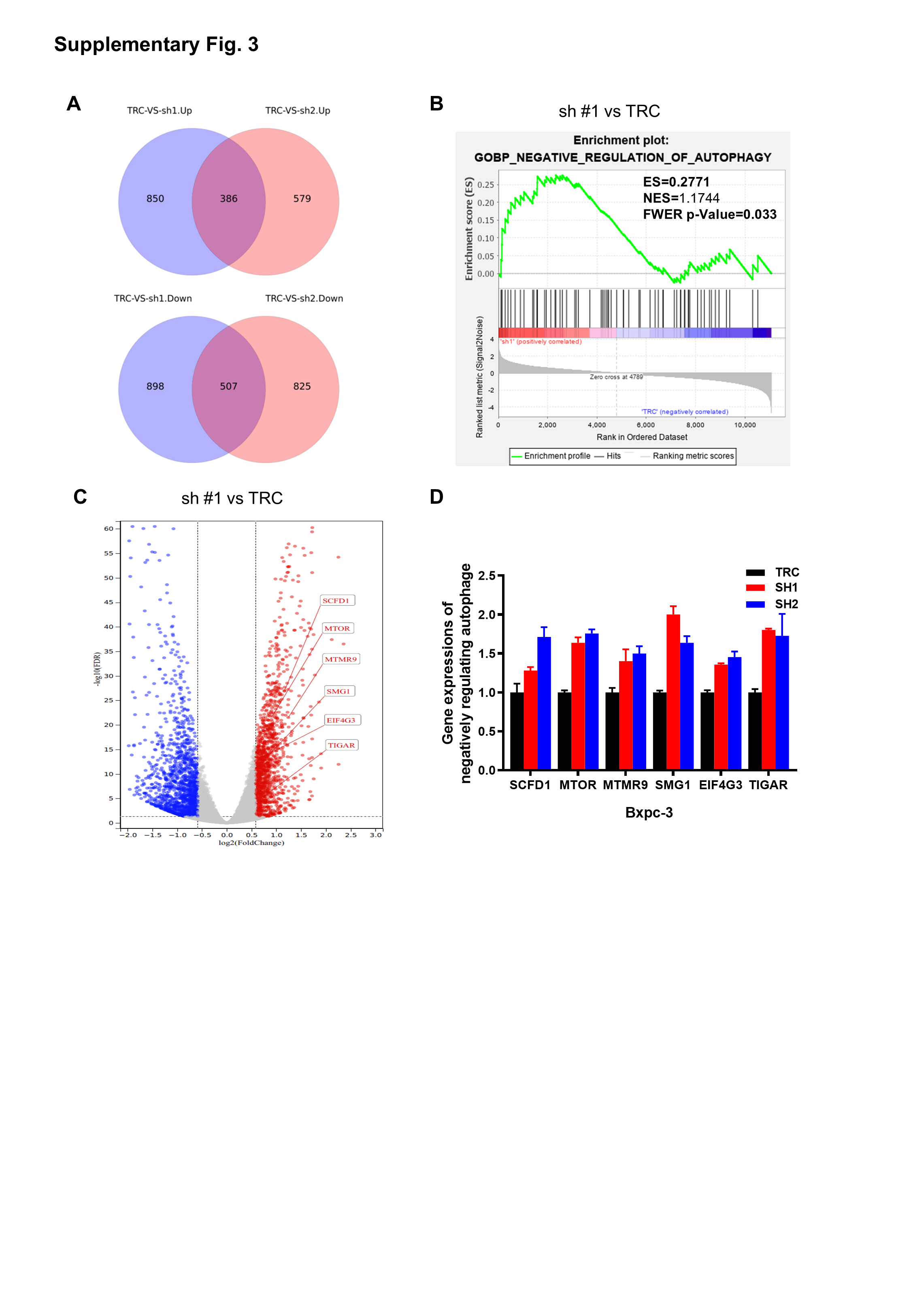

Supplement: Supplementary file 4 — supplementary Figure 3 [file 41419_2026_8915_MOESM4_ESM.tif]

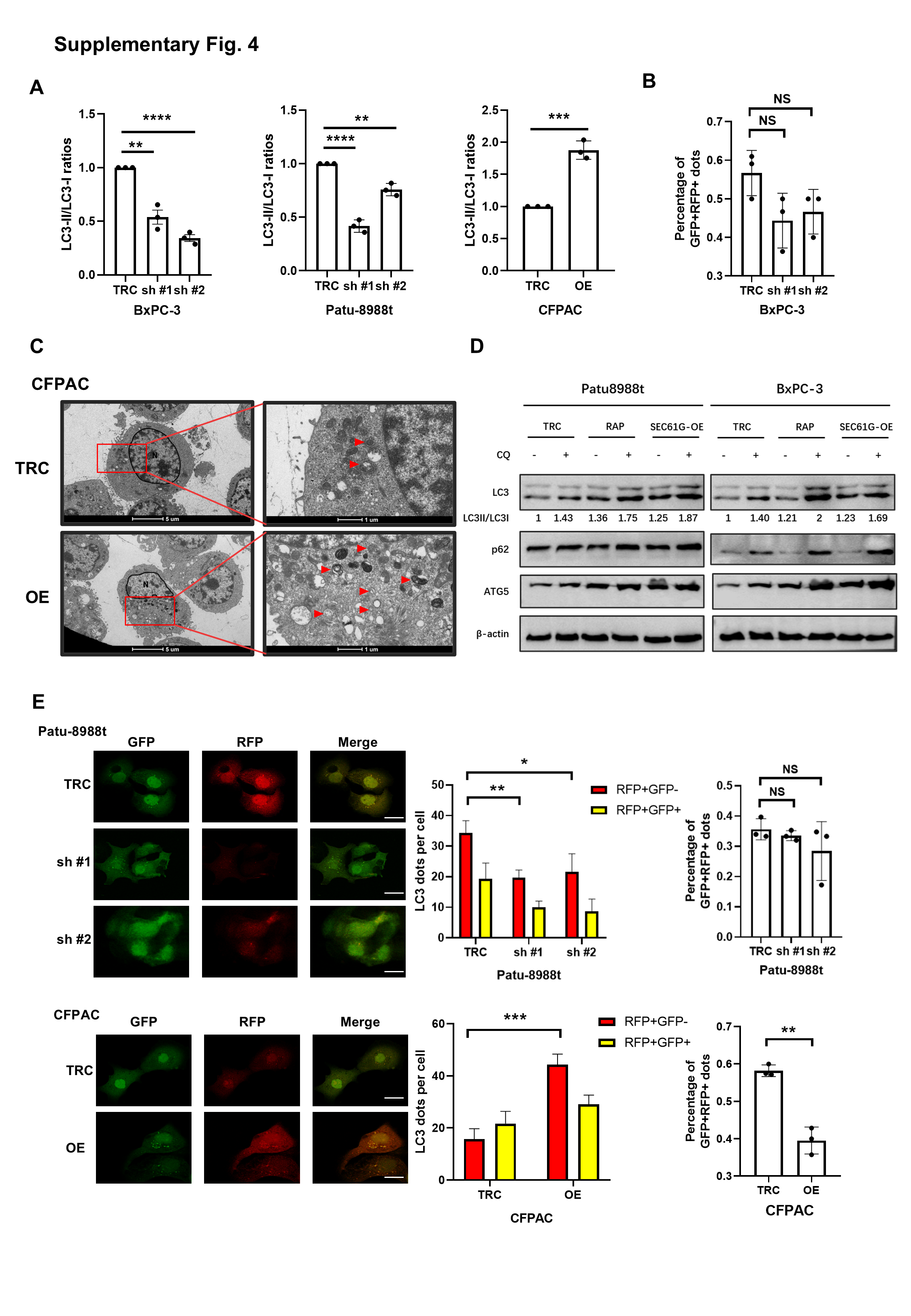

Supplement: Supplementary file 5 — supplementary Figure 4 [file 41419_2026_8915_MOESM5_ESM.tif]

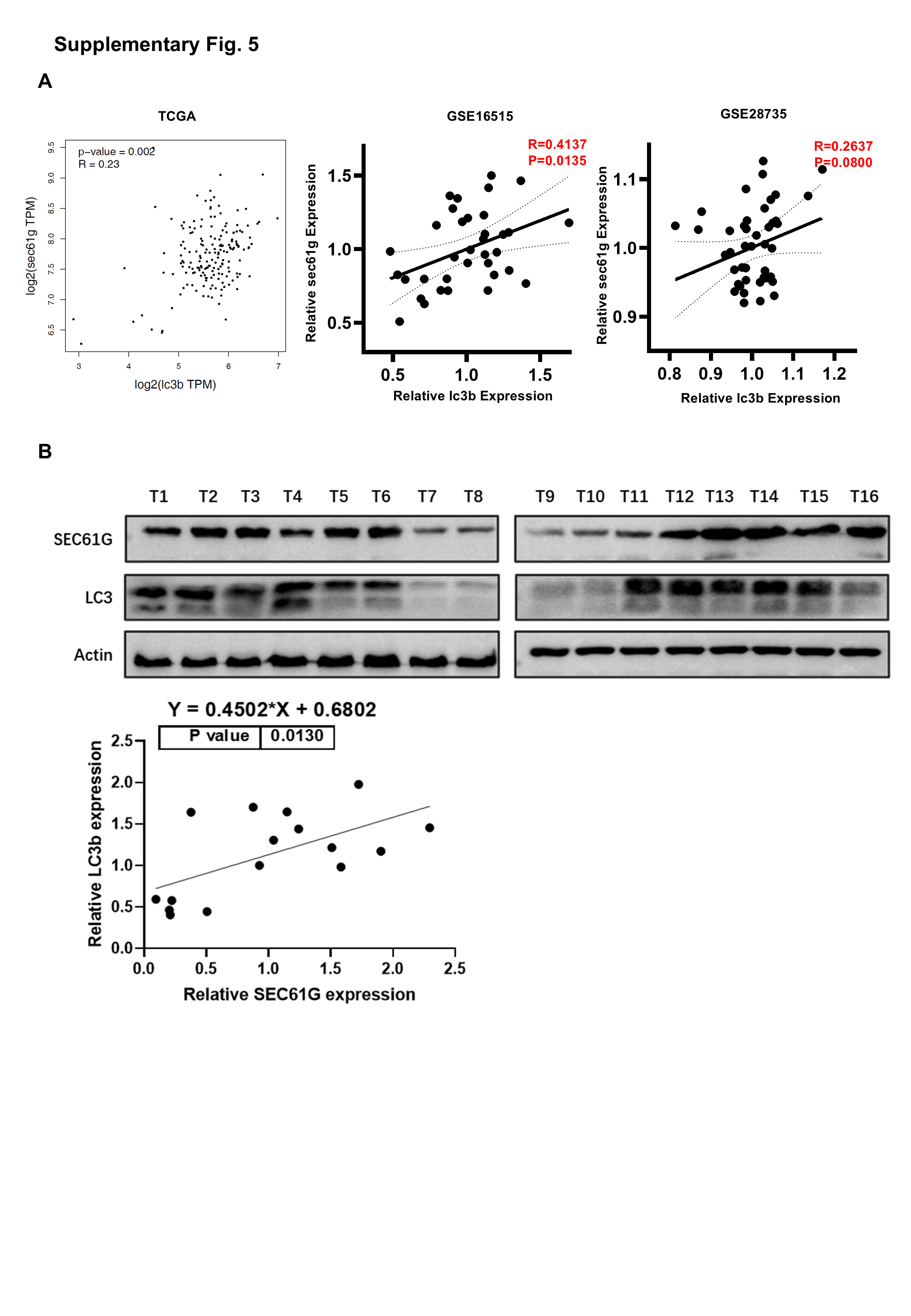

Supplement: Supplementary file 6 — supplementary Figure 5 [file 41419_2026_8915_MOESM6_ESM.tif]

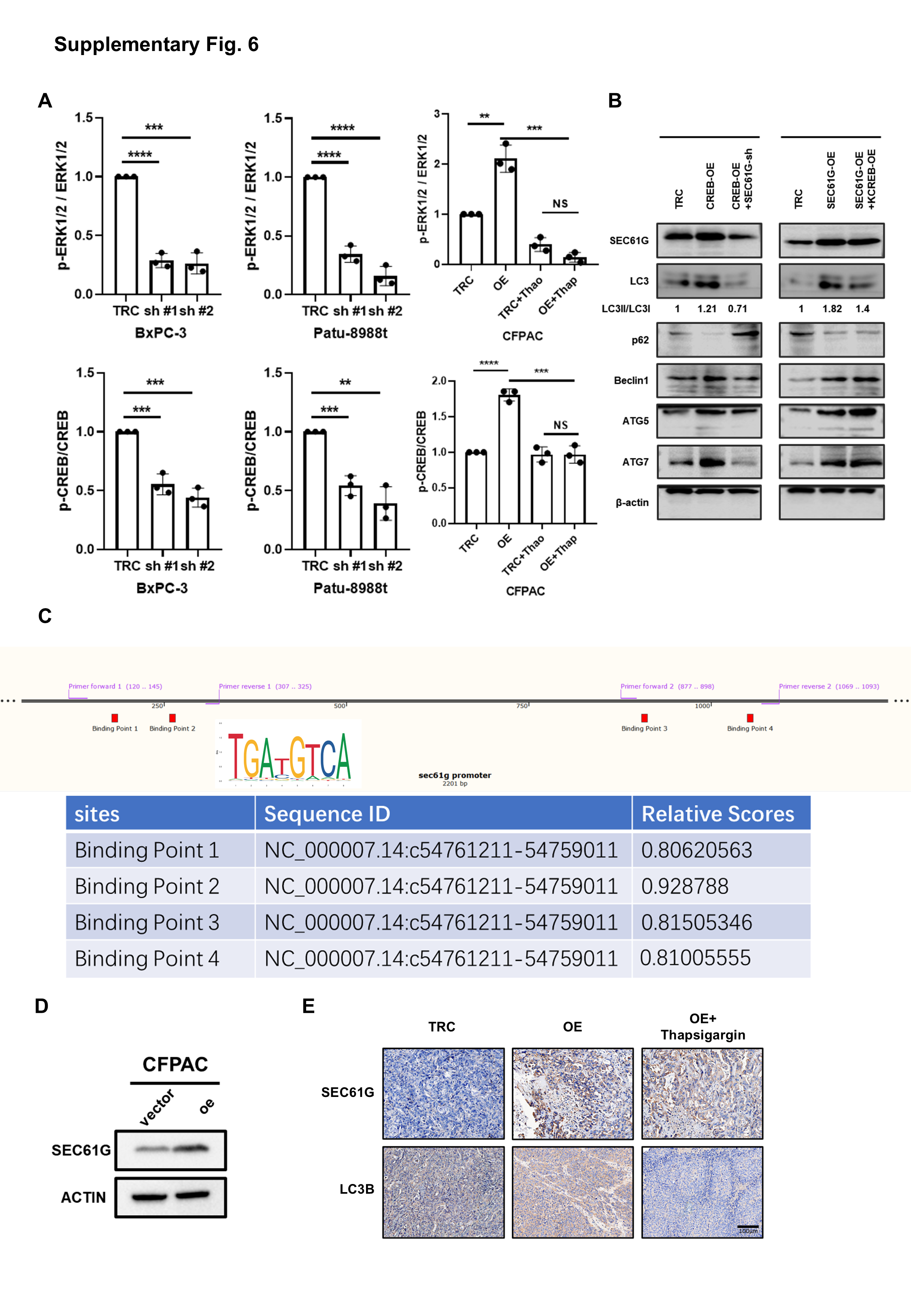

Supplement: Supplementary file 7 — supplementary Figure 6 [file 41419_2026_8915_MOESM7_ESM.tif]
