# Supplementary material for: CREB-SEC61G feedback loop sustains enhanced autophagy and boosts proliferation in PDAC
Source: Cell Death Dis. 2026 May 29;17(1):669. doi: 10.1038/s41419-026-08915-7 (PMC13424619; doi:10.1038/s41419-026-08915-7)

# Uncropped original western blots

Fig.1D

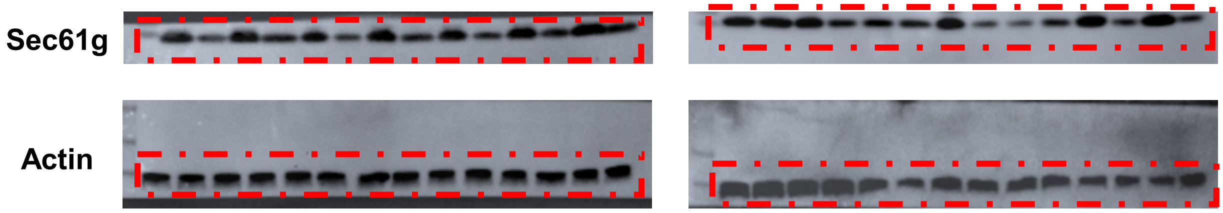


Fig.2A

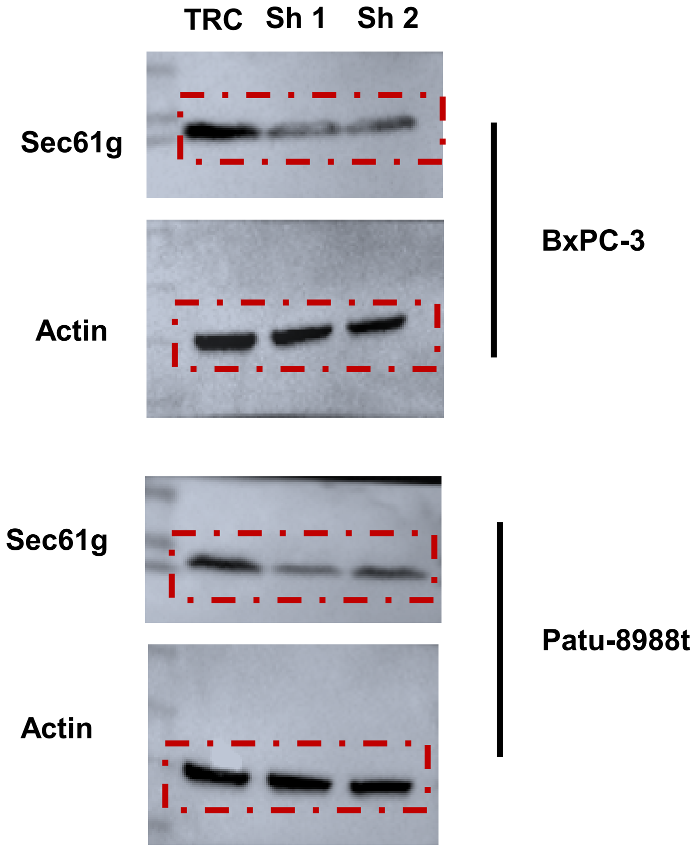

Fig.5A

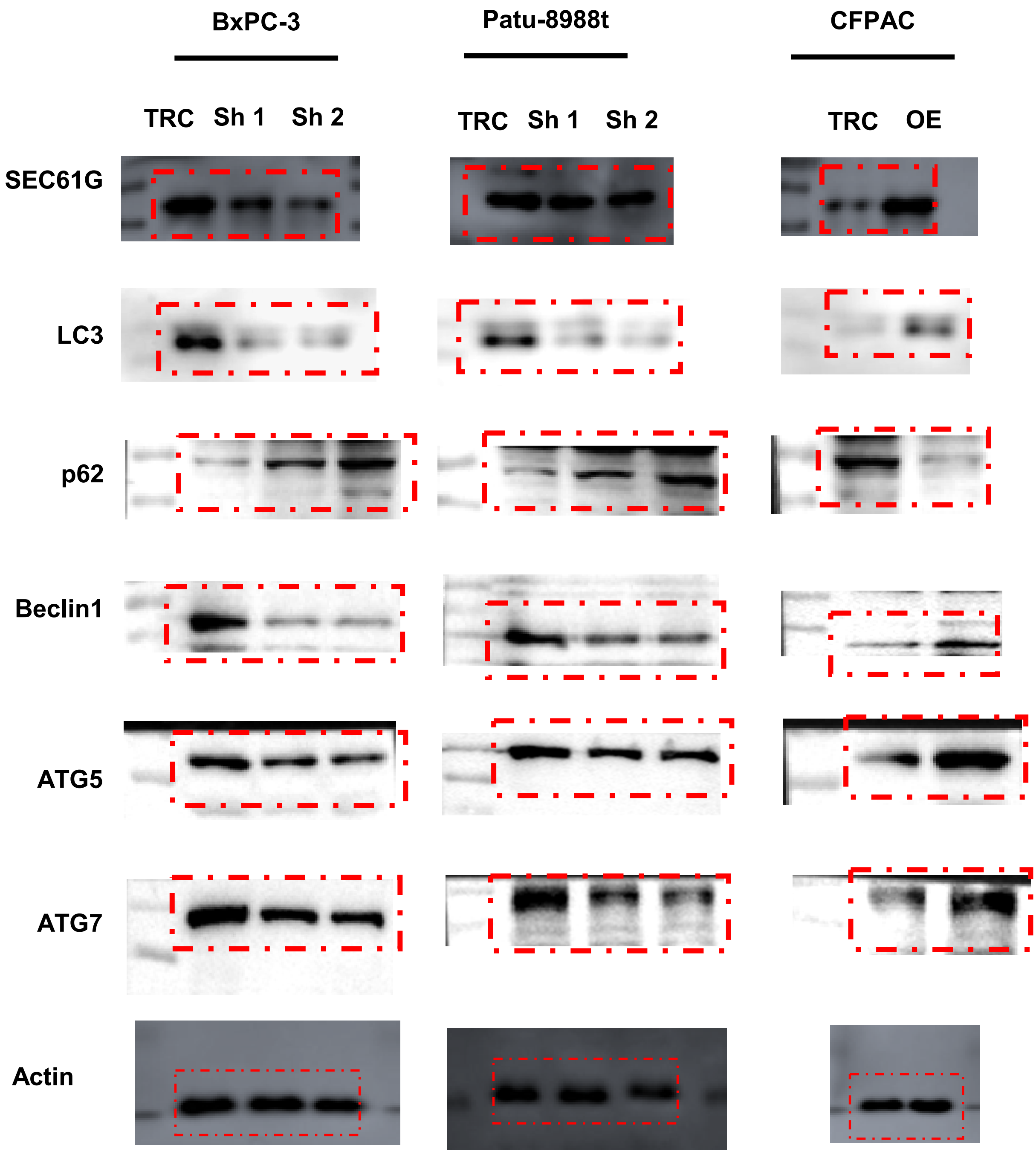
BxPC-3
Patu-8988t
CFPAC
TRC Sh 1 Sh 2
TRC Sh 1 Sh 2
TRC OE
SEC61G
LC3
p62
Beclin1
ATG5
ATG7
Actin

Fig.6B

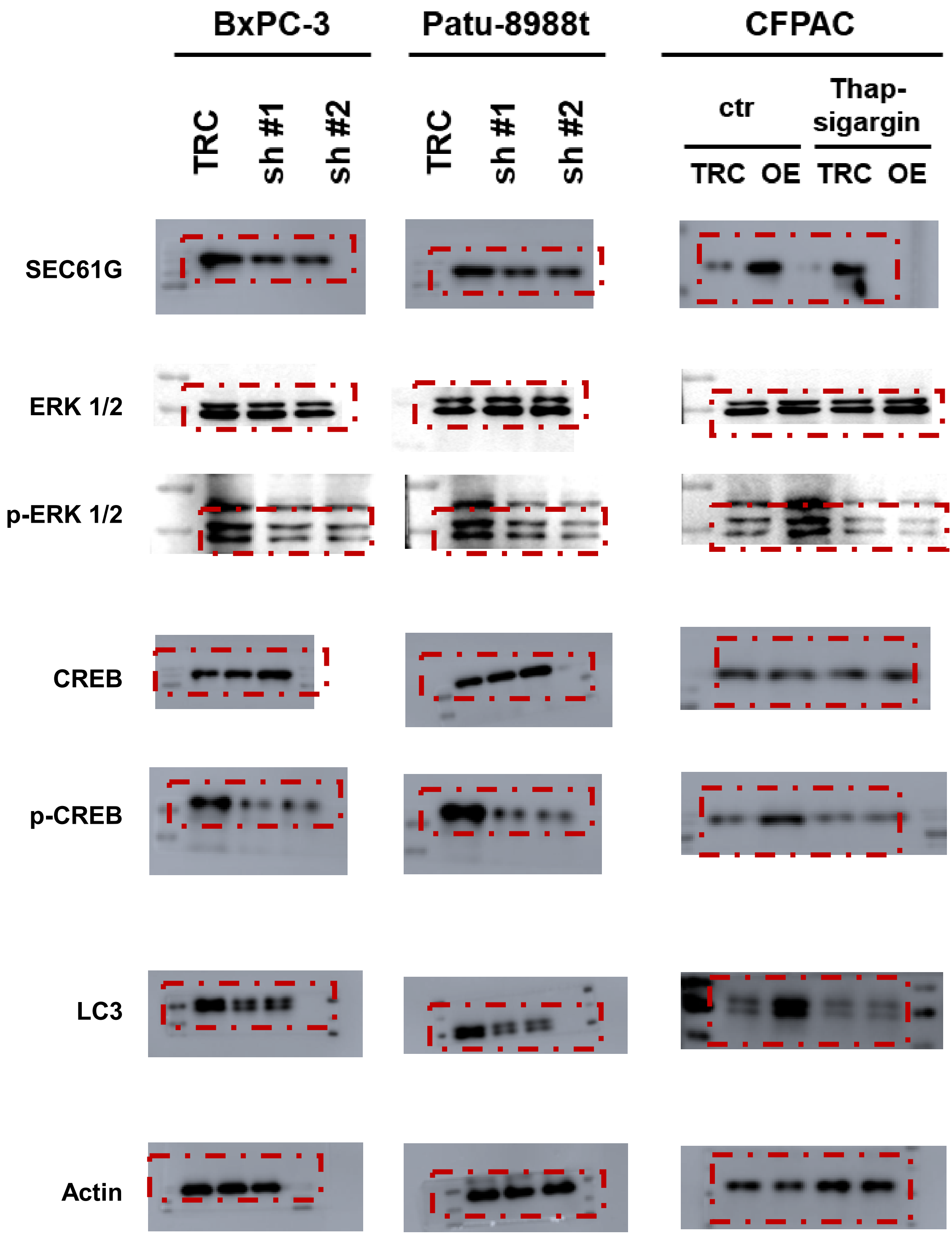
BxPC-3
Patu-8988t
CFPAC
TRC
sh #1
sh #2
TRC
sh #1
sh #2
ctr
Thap-
sigargin
TRC OE TRC OE
SEC61G
ERK 1/2
p-ERK 1/2
CREB
p-CREB
LC3
Actin

Fig.6C

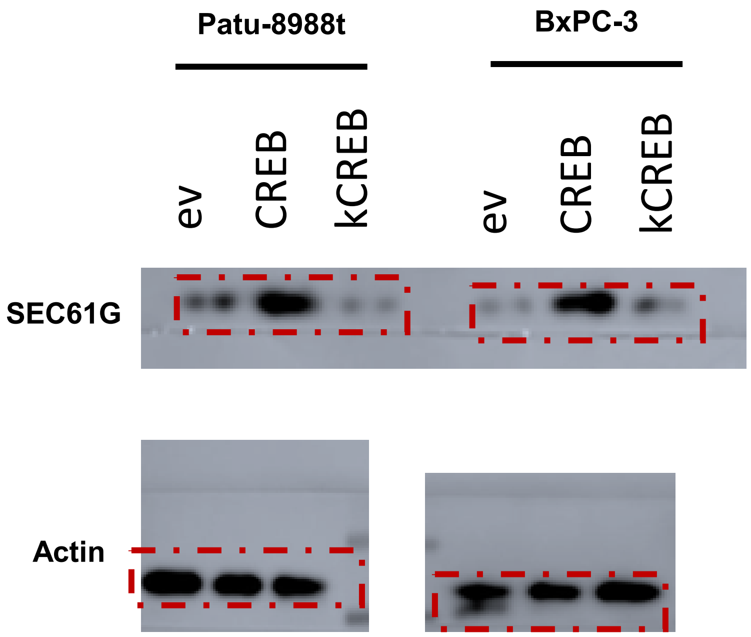
Patu-8988t
BxPC-3
ev
CREB
kCREB
ev
CREB
kCREB
SEC61G
Actin

Fig.7K

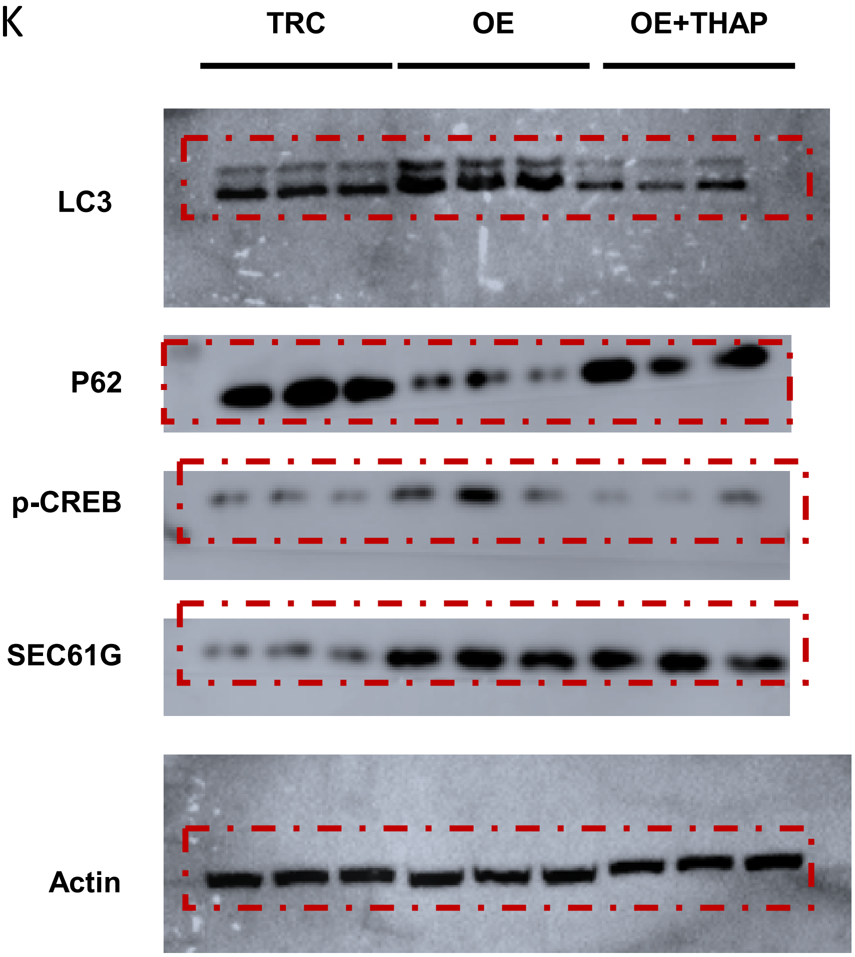
TRC
OE
OE+THAP
LC3
P62
p-CREB
SEC61G
Actin

Fig.S2A

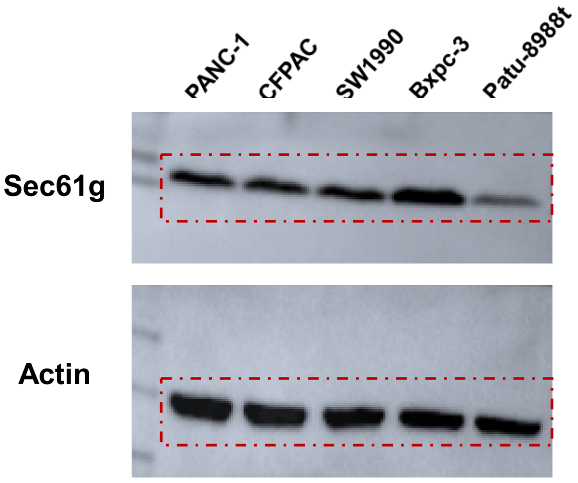


Fig.S2E

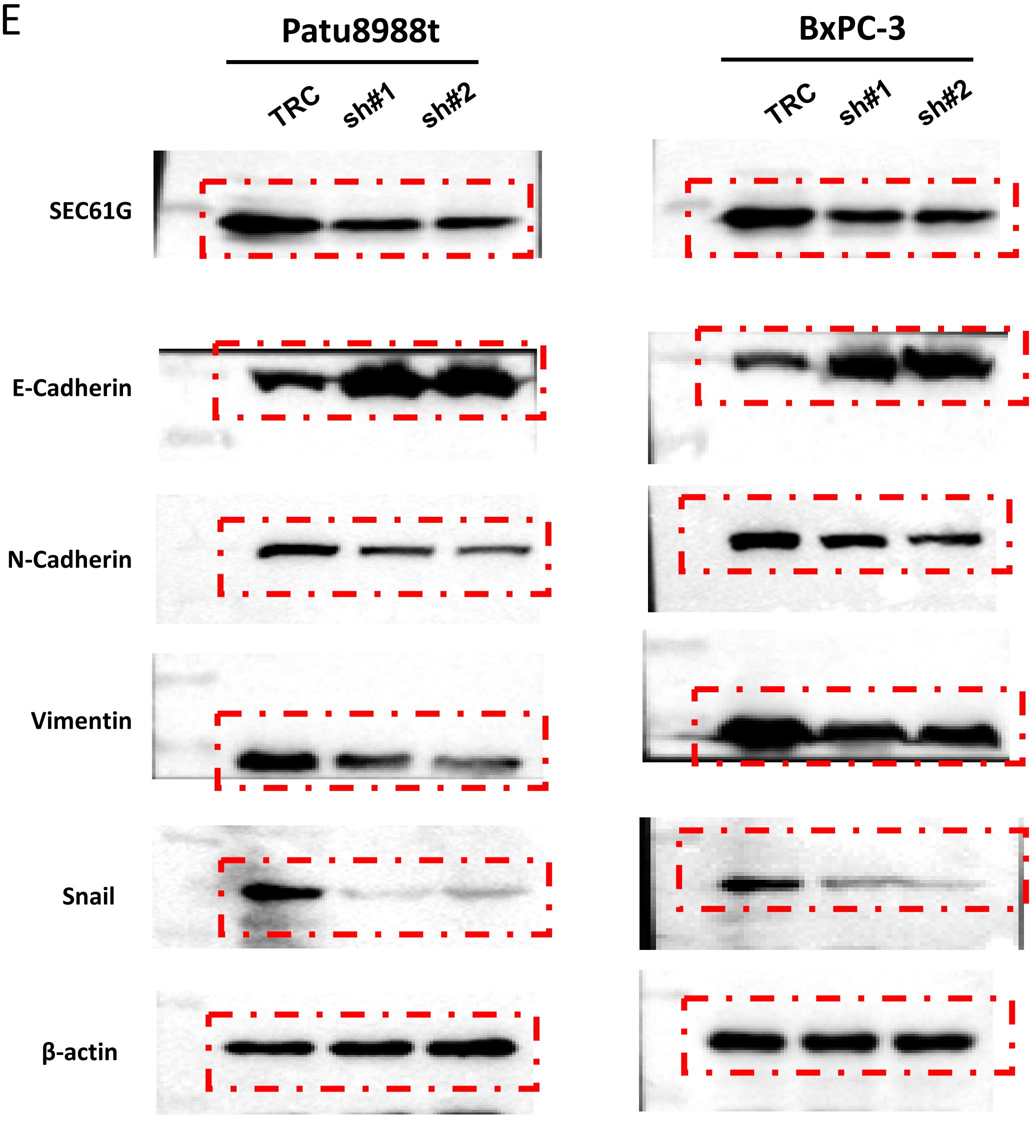

Fig.S4D

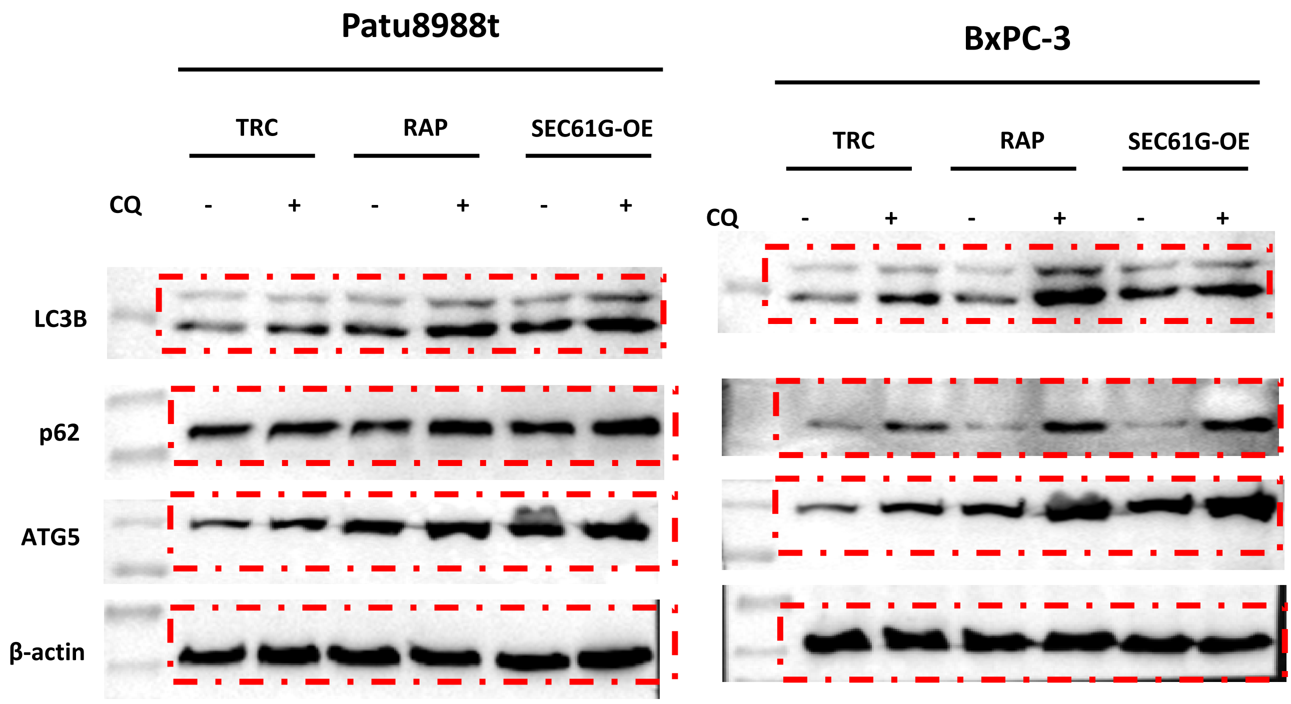


Fig.S5B

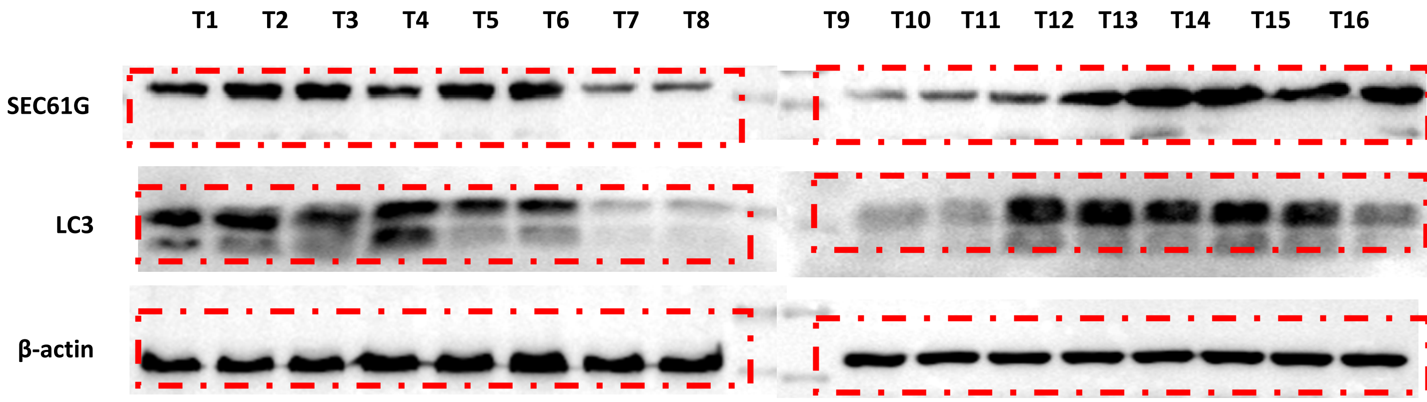

Fig.S6B

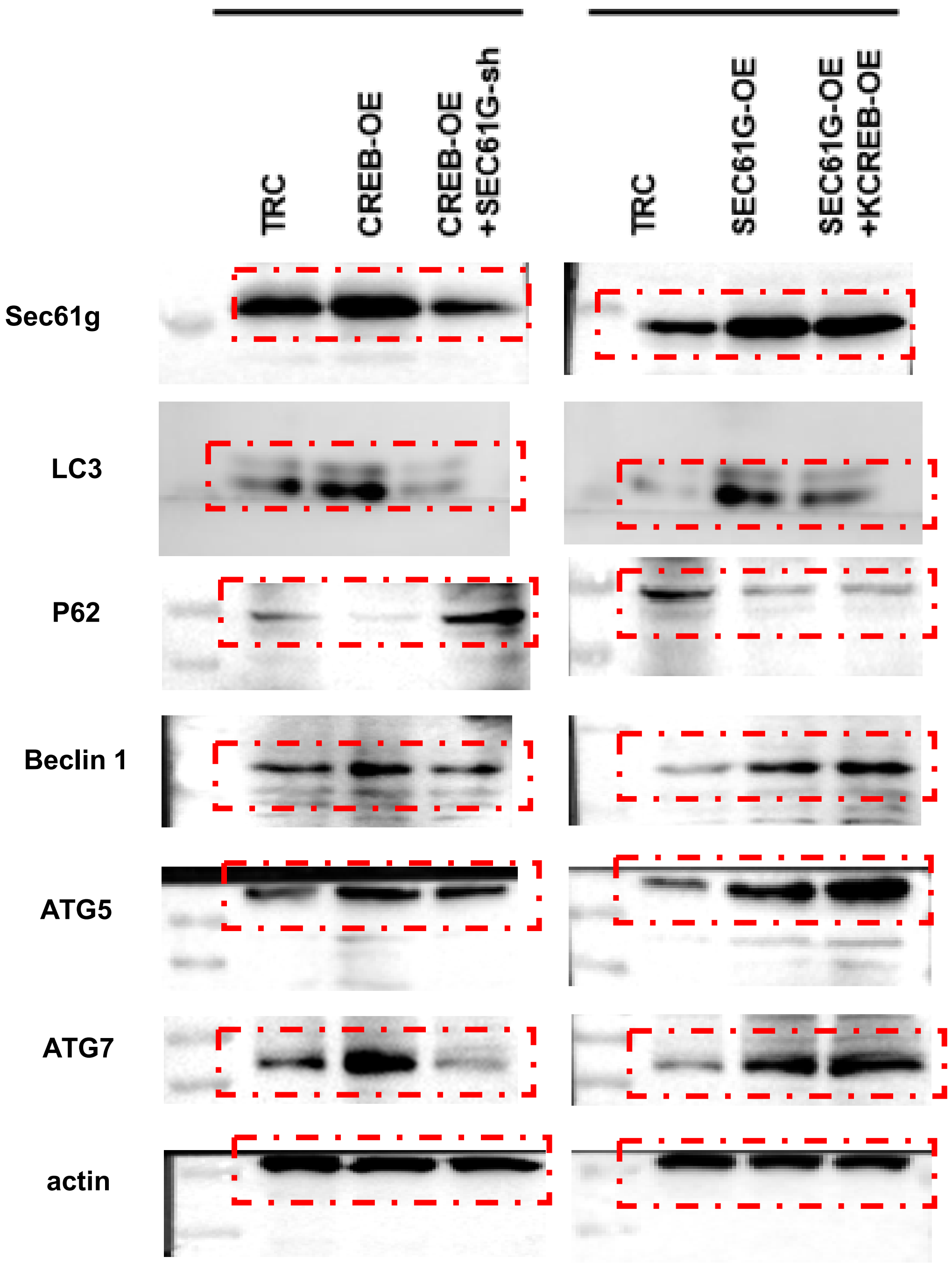


Fig.S6D

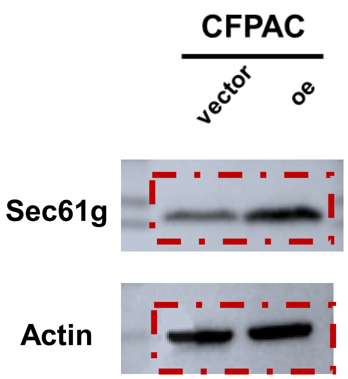

Supplement: Supplementary file 8 — uncropped original western blots [file 41419_2026_8915_MOESM8_ESM.pdf]
